# Global Transcriptome Analysis of the Scorpion *Centruroides noxius*: New Toxin Families and Evolutionary Insights from an Ancestral Scorpion Species

**DOI:** 10.1371/journal.pone.0043331

**Published:** 2012-08-17

**Authors:** Martha Rendón-Anaya, Luis Delaye, Lourival D. Possani, Alfredo Herrera-Estrella

**Affiliations:** 1 Laboratorio Nacional de Genómica para la Biodiversidad, Centro de Investigación y de Estudios Avanzados (CINVESTAV), Irapuato, Guanajuato, México; 2 Departamento de Ingeniería Genética, Centro de Investigación y de Estudios Avanzados (CINVESTAV), Irapuato, Guanajuato, México; 3 Departamento de Medicina Molecular y Bioprocesos, Instituto de Biotecnología, Universidad Nacional Autónoma de México (UNAM), Cuernavaca, Morelos, México; University of North Carolina at Charlotte, United States of America

## Abstract

Scorpion venoms have been studied for decades, leading to the identification of hundreds of different toxins with medical and pharmacological implications. However, little emphasis has been given to the description of these arthropods from cellular and evolutionary perspectives. In this report, we describe a transcriptomic analysis of the Mexican scorpion *Centruroides noxius Hoffmann*, performed with a pyrosequencing platform. Three independent sequencing experiments were carried out, each including three different cDNA libraries constructed from RNA extracted from the whole body of the scorpion after telson removal, and from the venom gland before and after venom extraction. Over three million reads were obtained and assembled in almost 19000 isogroups. Within the telson-specific sequences, 72 isogroups (0.4% of total unique transcripts) were found to be similar to toxins previously reported in other scorpion species, spiders and sea anemones. The annotation pipeline also revealed the presence of important elements of the small non-coding RNA processing machinery, as well as microRNA candidates. A phylogenomic analysis of concatenated essential genes evidenced differential evolution rates in this species, particularly in ribosomal proteins and proteasome components. Additionally, statistical comparison of transcript abundance before and after venom extraction showed that 3% and 2% of the assembled isogroups had higher expression levels in the active and replenishing gland, respectively. Thus, our sequencing and annotation strategies provide a general view of the cellular and molecular processes that take place in these arthropods, allowed the discovery of new pharmacological and biotechnological targets and uncovered several regulatory and metabolic responses behind the assembly of the scorpion venom. The results obtained in this report represent the first high-throughput study that thoroughly describes the universe of genes that are expressed in the scorpion *Centruroides noxius Hoffmann*, a highly relevant organism from medical and evolutionary perspectives.

## Introduction

For decades, the study of venomous animals has focused on the isolation and biochemical characterization of specific venom components that have medical or biotechnological importance. Indeed, scorpions have been extensively studied under this optic, which has lead to the identification of hundreds of different transcripts encoding toxic peptides, and peptides with neurotoxic activities that are important not only in medical terms, but have been used as pharmacological tools to understand the function of different ion channels [1]. However, scorpions are not only interesting organisms only because of their toxin diversity, but also because they represent the most ancient terrestrial animals that fossil records have identified. About 2000 species have been described around the world, which also implies that scorpions are extremely well adapted arthropods that have managed to survive in different environmental conditions. Buthidae, the largest family of extant scorpions, has been proposed to be one of the most ancient or basal families [2]. Cladistic and phylogenetic analysis suggested that they arose ∼350 million years ago [3], [4] and, after a physical separation upon the partition of the Africa-South America continents (∼150 My ago), several speciation events gave rise to different genus such as *Buthus, Mesobuthus, Parabuthus, Hottentotta, Leiurus* and *Androctonus* in Africa and Asia, and *Tityus* and *Centruroides* in South and North America, respectively [5]. In Mexico, the medically important scorpions that belong to the genus *Centruroides* are distributed along the states of the Pacific coast; among these, *Centruroides noxius Hoffmann* (from and then, simply abbreviated *C. noxius*) has been identified as the most dangerous species in the country (LD_50_ of 3.8 µg venom per 20 g mouse wt) [6]. In this context, it becomes clear that at the molecular and morphological levels, the venom glands of these arthropods have evolved over the past 400 million years giving as a result, a complex toxic arsenal that is efficiently used for prey and defence. Unfortunately, the evolutionary relevance of scorpions has been scarcely analyzed over the years.

Different “omic" approaches have become a very powerful strategy for understanding the complexity of venomous animals; transcriptomics in particular, has been widely used to explore the transcriptional diversity of venom glands of several scorpion species. Except for the analysis of ESTs derived from “resting" or “replete" venom glands of the buthid scorpion *Hottentota judaicus* [7], the rest of the reported transcriptomes of scorpions from the Buthidae, Scorpionidae, Euscorpiidae, Caraboctonidae, Liochelidae and Iuridae families have shared a methodological principle: the RNA was collected from the venom glands 2 to 5 days after venom extraction by electric stimulation, implying that the gland is engaged in regenerating its venom, which can be referred to as “active" or “replenishing" state [8]–[12]. The high proportion of toxin-like transcripts identified in these reports (from 30 to 78% of the total ESTs) has emphasized the specialized role of the venom glands in the production of toxin peptides, unfortunately, the small number of tags generated by Sanger sequencing as well as the use of telson-specific RNA for random EST screening have restricted the identification of other cellular processes and molecular dynamics that take place in these organisms (reviewed by [13]). In addition to these limitations, little scorpion genomic information is available: the genome size, gene content, intron/exon complexity and the genomic intra-inter species variation are important aspects that remain unknown. Under these circumstances, it becomes essential to consider new sequencing platforms that have been proved to be excellent tools in the study of the transcriptional and genomic universe of non-model organisms. Indeed, new sequencing technologies allow us not only to measure in qualitative terms the diversity of genes (genomic DNA or ESTs), but they also give us the possibility of making quantitative comparisons of the transcriptional profiles under different conditions, tissues or treatments.

In this report, we take advantage of the qualitative and quantitative capacities of the 454-pyrosequencing platform in order to conduct a global transcriptomic analysis of the Mexican scorpion *C. noxius* that aimed to understand important biological and evolutionary aspects of these ancestral arthropods. The analysis included the construction of cDNA libraries from different origins (body and telson under two different treatments), a *de novo* assembly that provided *bona fide* arthropod scaffolds, a general annotation of the assembled sequences, the identification of toxin families, a phylogenetic reconstruction that aimed to get new insights into the evolution of this species, and the statistical comparison of the transcriptional abundance of different genes before and after venom extraction.

## Materials and Methods

### cDNA Library Construction

Nine cDNA libraries were constructed for this analysis. Three of them with RNA extracted from the body after telson removal of a single *C. noxius* scorpion, and the rest of the libraries with RNA extracted from the telson of 20 individuals in two different conditions: in an active state of the venom gland (the telson was removed five days after venom extraction with electric stimulation) and replenishing state (no milking was performed before telson removal). Total RNA was extracted with TRIZOL (Invitrogen). cDNA synthesis was performed with 3.5 µg of total RNA using Message Amp-II kit (Ambion) following the protocol as recommended by manufacturers. The first strand cDNA synthesis was primed with T7 oligo(dT) primers. After a second strand cDNA synthesis reaction, 5–10 ng of synthesized double stranded cDNA were amplified by in vitro transcription and the resulting 5–7 µg of antisense RNA (aRNA) were purified using Qiagen RNAeasy columns (Qiagen). A second round of cDNA synthesis was performed using the aRNA as template. First and second strand cDNA synthesis were as described above except that random nonamers (Amersham) were used at the first strand synthesis stage. This procedure yielded about 4 µg of cDNA that were purified using the DNA Clear Kit for cDNA purification (Ambion). cDNA was nebulyzed to obtain fragments of 200–700 bp before sequencing.

### 454 Sequencing and Assembly

Approximately 3 µg of sheared cDNA of each library were used for 454 sequencing. The cDNA samples were end-repaired and adapter ligated according to [14]. Streptavidin bead enrichment, DNA denaturation and emulsion PCR were also done according to procedures previously described [14]. Three independent sequencing runs were performed using the GS20, GS-FLX and FLX-Titanium systems. Each of them included three cDNA libraries, one from the body without telson, one from active telsons and the third one from resting telsons. Altogether, these runs resulted in a total number of 3 008 049 reads of variable lengths, spanning form 100 up to 450 bp. Unique identifiers were assigned to the reads according to the library and 454 system from which they were obtained. Raw sequencing data are archived under accession number SRP010317 in the NCBI Sequence Read Archive (SRA, http://www.ncbi.nlm.nih.gov/Traces/sra). Accession codes by 454 sequencing system are SRX115875.4; SRX115901.2 and SRX115902.2. A global *de novo* assembly of the reads was performed using Newbler 2.5 with the default parameters for EST analysis.

### Qualitative Analysis of the Assembled Sequences

The assembled isotigs were blasted against NCBI-NR, the *Drosophila melanogaster* protein collection reported in FlyBase and the toxins deposited in ToxProt (http://www.expasy.ch/sprot/tox-prot/tox-prot_stat.html). The cut-off criteria were: e-value <1e−04, identity percentage >30% and coverage >30%. HMMER and SignalP were used to find conserved protein domains and signal peptides for the putative venom secreted components. The blastp outputs were also used to obtain the gene ontologies with Blast2Go [15].

The collection of hairpin precursors and mature microRNAs (www.mirbase.org) was formatted into a nucleotide database in order to look for small non-coding RNAs within the assembled transcripts and singlets using blastn. The cut-off criteria were set as follows: e-value <1e−02 and identity percentage >80% and hairpin precursor coverage >35%.

A taxonomic profile of the transcriptome was obtained with MEGAN [16], [17] in order to quantify the number of arthropod non-specific assembled sequences.

### Phylogenomics

Toxin-like isotigs were aligned with other toxin peptides from different scorpion species using Clustalw [18]. The corresponding phylogenies were constructed with Maximum Likelihood (PhyML [19]), running 1000 bootstrap replicates.

Two different sets of peptide sequences of coding eukaryotic genes were constructed: (1) 150 genes from 27 distant eukaryotic species; (2) 60 genes from 59 arthropod species (the GIs of the arthropod coding sequences were taken from Rieger and collaborators [20]). Individual genes were aligned with MUSCLE [21] and low quality regions removed using GBlocks with default parameters [22]. The resulting protein alignments were concatenated to generate a single amino acid matrix for each data set that, for the eukaryotic group consisted of 23,685 amino acid positions and 9,926 for the arthropod specific group. The phylogenies of individual and concatenated orthologues were constructed with PhyML [19], performing 1000 bootstrap replicates and using the LG and JTT models, respectively, which were selected after an evaluation of the amino acid alignments with ProtTest [23].

### Statistical Analysis

The reads obtained after the pyrosequencing runs can be easily discriminated by treatment (active/resting telson) using the identifiers they were assigned, which makes it possible to calculate the exact number of reads from each cDNA library that overlap in the isotigs. Since the isotigs are considered by the assembler as splicing variants of a single gene, it was necessary to quantify the number of reads per isogroup to avoid redundancy and overestimation of the actual read content for both telson conditions. Therefore, all the reads aligning to each isogroup were summarized and introduced into a matrix that was used as a contingency table to perform a Fisher test using R. The resulting p-value measures the minimum false positive rate that is incurred when calling that test significant. In a similar way, the q-value gives each hypothesis test a measure of significance in terms of a certain error rate. However, the q-value measures the minimum false discovery rate that is incurred when calling that test significant, which means that it measures the expected proportion of false positives among the tests previously found to be significant. Because of this important difference, the p-values obtained after the Fisher test were evaluated with the R module “QVALUE" [24].

## Results and Discussion

### Sequencing and Assembly

The global assembly using Newbler 2.5 resulted in 26,672 isotigs, grouped in almost 19,000 isogroups (Table 1) that represent over 80% of the total reads obtained after 3 runs of 454 pyrosequencing. The average length of the isotigs was 950 nt, and 19,543 of them were >500 bp long. It is also interesting to notice that 93% of the assembled isotigs had open reading frames >100 nt long, which translates into 16,823 isogroups (89%) or unique genes with ORFs. The remaining 7% of the isotigs that did not show predicted ORFs was also analyzed. The average length and depth of this type of isotigs is 268.5 bp and 18.4 reads, respectively, which shows that these are short sequences with low coverage. Intriguingly, around 1% of these transcripts were read several times (from 100 up to 10,000 reads deep) in the telson and the body of the scorpion and thus, they represent putative non-coding long RNAs. Unfortunately, until now no common structural features have been identified in long non-coding RNAs. Some other ESTs without predicted ORFs could also be artefactual sequences and 351 isotigs could even be considered chimeric splicing variants since they belong to isogroups that contain isotigs with open reading frames.

**Table 1:** Assembly statistics.

Raw sequences	
Resting telson	1 249 489
Active telson	981 028
Body	777 532
Total Reads	3 008 049
**Assembly**	
Assembled	2 477 532
Singlets	424 134
Repeats	3 894
**Isotigs**	**26 672**
avgContigCnt	2.1
largestContigCnt	16
numberWithOneContig	16 257
avgIsotigSize (bp)	950
N50IsotigSize (bp)	1 287
**Isogroups**	**18 979**
avgIsotigCnt	1.4
numberWithOneIsotig	15 557

In biological terms, thinking of the isogroups as unique genes and taking into account the number of genes identified in *Drosophila melanogaster* and *Ixodes scapularis* (15 and 25 thousand, respectively), we might be close to the range of arthropod gene content, although this number will have to be validated in future studies. On the other hand, the biological interpretation of the number of isotigs and isogroups leads to the observation that 39% of the assembled sequences are composed by more than 2 contigs or exons, and that surprisingly only 17% of the isogroups have splicing variants. It has been reported that 70–95% of the human genes undergo alternative splicing [25] and almost 40% of the fruit fly genes are spliced at early developmental stages [26]. Therefore, these splicing events in *C. noxius* seem to be abnormally small. The actual number of genes with splicing variants will have to be analyzed once genome data are available and the intron/exon content quantified.

Over 400 thousand reads (14% of the total amount of produced sequences) were classified as singlets after the global assembly (Table 1). Eighty five percent of these sequences correspond to telson specific reads and most of them (50% of the total number of singlets), were generated with the GS20 system. The singlet average length is 127 (±103) bp and 53% of them are <100 bp long. This high proportion of short singlets can be due to sequencing artefacts that could result in low quality sequences or might also imply that the coverage of telson ESTs has not reached an optimal level and thus, there are still unassembled short transcripts that could in fact have gland specific expression since they did not overlap with body extracted reads.

Having cDNA libraries constructed with RNA from different origins, we were able to discriminate between genes that are specifically expressed in the telson, the body or ubiquitously, by analysing the read composition of the assembled isotigs. Most of the transcripts (73%) are present in both, the telson and the body of the scorpion, whereas only 3,5% of the isotigs showed body specific expression and 23,8% telson specific expression.

### Transcript Annotation

**Figure 1:**
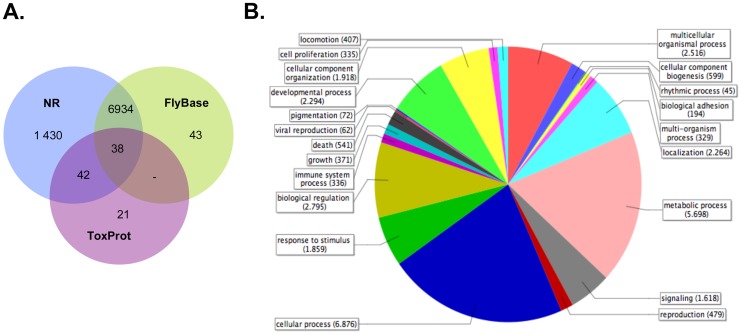
Homology based annotation. The isotigs were blasted against NCBI-NR, Flybase and ToxProt (eval <1e−05, identity >30%, coverage >30%); the intersection of the significant hits is shown in A. Gene Ontologies were also obtained using Blast2Go; general biological processes represented in the transcriptome of *C. noxius* are shown in the legend of chart in B.

The annotation strategy using NCBI-NR, the *D. melanogaster* protein collection and the toxin peptides from ToxProt, revealed that 51% of the isogroups with open reading frames had significant hits in at least one of the databases (Fig. 1A). Thus, it is likely that there is a significant number of scorpion-specific genes that had not been discovered so far. The fact that such a large proportion of sequences had no significant similarities in the databases might also be due to intergenic or pervasive transcription, which has been widely observed in other eukaryotes [27], [28].

Among the annotated transcripts, three important components of the microRNA processing machinery, Dicer, Drosha and Argonaute (Ago), were identified. Two isogroups showed 80% identity to dicer-1 from *I. scapularis* and aligned in two different regions at the C-terminus, which may correspond to two partial sequences of the same transcript that do not contain overlapping sequences. The same situation was observed for Drosha, in which two isogroups covered 90% of the protein sequence but could not overlap because of the lack of a 66 bp sequence. Additionally, two isogroups showed significant similarity to Ago proteins; one of them displayed 53% identity and 36% coverage of Ago1 (LD36719p from *D. melanogaster*), and the second one had 50% identity and 54% coverage of Ago3 from *H. sapiens*. Having detected some of the main components of this pathway, it was necessary to evaluate the presence of putative microRNAs in our collection of assembled transcripts and singlets. The isotigs without open reading frame, those with ORFs but no significant blast hits and the singlets were blasted against a database of hairpin precursors and mature microRNAs (http://www.mirbase.org/). Surprisingly, several potential candidates (3 isogroups and 93 singlets) with identity values >80% to different microRNAs from other species were identified. Notably, 84% of these sequences covered more than 60% of the stem loops and they also contained mature microRNAs within the hairpin positions (Table 2 and Table S1). Taken together, these results indicate that the use of microRNAs as a post-transcriptional control mechanism is widely distributed among eukaryotes, from basal or ancient clades up to recently diverged taxa.

**Table 2:** Putative microRNAs.

Scorpion Sequence Name	microRNA-hairpin	%id	e-value	hairpin coverage	mature microRNA	%id	Aln bp	e-value
GXU3QYM07H17KM	ppy-mir-566	87	4E−17	98,9	hsa-miR-5585-3p	100	17	4E−03
FXLSL4B01BPRPE	gga-mir-3533	86	2E−13	97,6	rno-miR-196c	100	14	7E−02
isotig03213	gga-mir-3533	86	1E−12	97,6	rno-miR-196c	100	14	4E−01
GXTJ2BI07H1UZ8	ppy-mir-1273	92	9E−30	91,3	hsa-miR-1273 g-3p	100	16	1E−02
GXU3QYM06G2YF3	ppy-mir-1273	93	5E−26	81,7	hsa-miR-1273f	95	19	7E−02
GXTJ2BI01ATJ35	hsa-mir-3927	98	3E−21	79,2	hsa-miR-3927	100	22	4E−06
ETFUWZZ02GED74	ptr-mir-566	86	3E−11	75,5	mml-miR-665	100	14	6E−02
					ptr-miR-665	100	14	6E−02
					hsa-miR-665	100	14	6E−02
EUKA23202H0XZD	isc-mir-96	89	9E−15	71,6	isc-miR-96	95	22	3E−04
					ppy-miR-96	95	21	1E−03
GXU3QYM05FQ6UF	gga-mir-3533	88	2E−11	69,4	rno-miR-196c	100	14	4E−02
EUKA23201B149O	isc-mir-96	87	1E−10	68,6	isc-miR-96	95	22	3E−04
					ppy-miR-96	95	21	1E−03
					eca-miR-96	95	21	1E−03
GXTJ2BI03DMAEW	ppy-mir-1273	86	1E−10	68,3	hsa-miR-1273e	100	16	1E−02
					bfl-miR-2013	100	13	8E−01
EUKA23202JL9NO	ppy-mir-1273	93	1E−22	67,3	hsa-miR-1273 g-3p	94	18	6E−02
EUC08LD01C4EUE	mmu-mir-1905	87	8E−09	66,3	mcv-miR-M1-3p	100	14	7E−02
GXTJ2BI08JJID5	mmu-mir-1905	87	7E−09	66,3	mcv-miR-M1-3p	100	14	5E−02
isotig06511	isc-mir-275	91	3E−13	62,7	nlo-miR-275	96	23	4E−04
					ngi-miR-275	96	23	4E−04
					nvi-miR-275	96	23	4E−04
					isc-miR-275	96	23	4E−04
					dpu-miR-275	96	23	4E−04
					dya-miR-275	96	23	4E−04
					dwi-miR-275	96	23	4E−04

Fifteen scorpion sequences (isotigs and singlets) showing significant identity to hairpins and mature microRNAs are displayed in this table. For the complete list of microRNA candidates, see Table S1.

A functional mapping of the gene ontologies against the KEGG metabolic pathways revealed that, in qualitative terms, most of the eukaryotic metabolic processes were identified in the transcriptome of *C. noxius*. These processes are particularly represented by isotigs of ubiquitous expression, body specific isotigs and singlets.

Since the body specific RNA was obtained from a macerate of the whole scorpion, the cDNA libraries could potentially include some bacterial or viral sequences from symbiotic or parasitic organisms. Therefore, knowing the proportion of non-specific scorpion sequences was an important control of the quality of the assembly. Even though some bacterial and viral sequences where identified, they were classified as singlets, which means that the assembled isotigs are *bona fide* eukaryotic sequences (Fig. S1). Although the cDNA construction was performed using oligo(dT) primers, it is possible that some of the non-eukaryotic sequences contain polyA tracts that were amplified in the first step of the cDNA synthesis. Some Actinobacteria, Flavobacteria, Firmicutes (*Bacillus* and *Clostridium*) and Proteobacteria were the most abundant clades, which is consistent with other reports where the same bacterial classes are present in the digestive tracts of insects and ticks [29], [30].

### Phylogenomic Analysis

**Figure 2:**
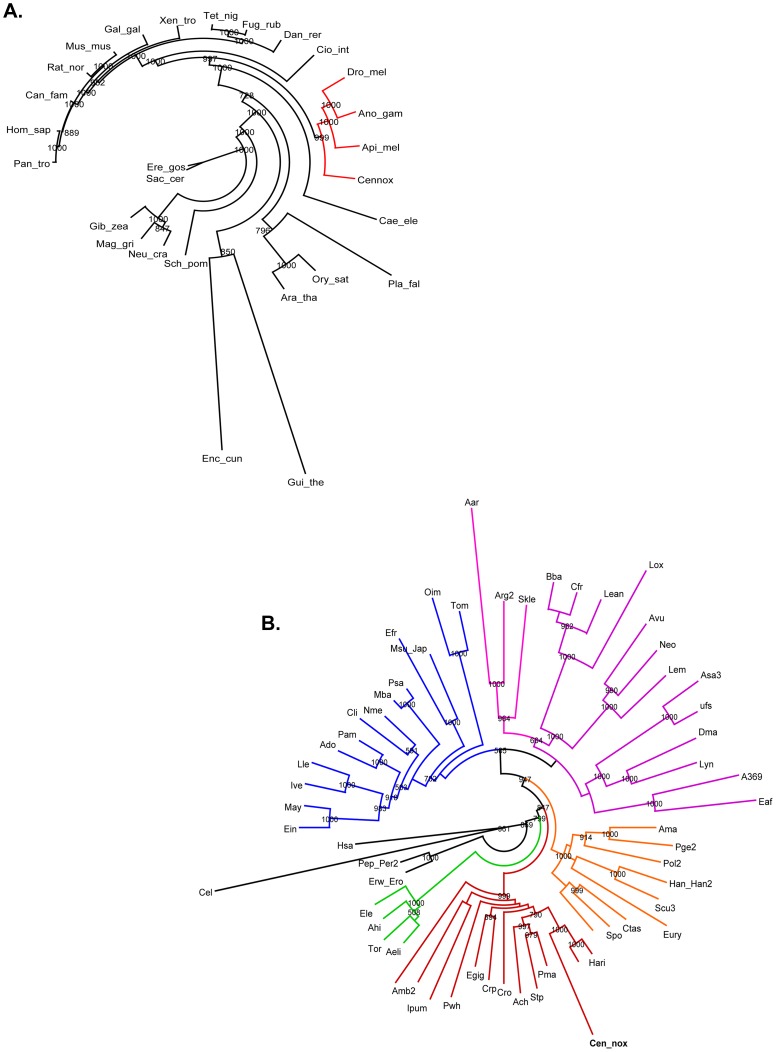
PhyML phylogenies with two independent amino acid datasets show long branch lengths for *C. noxius*. A. Tree topology of eukaryotic essential genes obtained from distant species. *C. noxius* is grouped with other arthropods (insects) as highlighted in red. B. Arthropod specific tree, where *C. noxius* is grouped with other Chelicerata (red), together with two scorpion species from Caraboctonidae and Scorpionidae families. Color code: blue, Hexapoda; purple, Crustacea; pink, Oligostraca; orange, Myriapoda; red, Chelicerata; green, Pycnogonida; black, outgroups (Onychophora; *H. sapiens* and *C. elegans*). See Table S2 and Table S3 for full names of the species. LG (A) and JTT (B) models were used, and 1000 bootstrap pseudoreplicates were performed. Bootrstrap support values >500 are shown in both trees.

Molecular phylogenies were traditionally constructed with ribosomal sequences, mitochondrial DNA or single orthologous genes, and they often yielded contradictory results. However, the concatenation of large sets of genes has been shown to reconstruct more accurately the evolutionary history of organisms [31]. The molecular phylogeny of Arthropoda in particular, has been difficult to resolve. Several attempts to overcome this incongruence have used different sets of orthologous genes, but the number of species considered for those analyses has also been limited. In this report, we used a collection of orthologous genes from 59 arthropod species amplified and analyzed by Regier and collaborators [20]. The concatenate of the supergene includes 58 different genes from Hexapoda, Myriapoda, Arachnida, Xiphosura and Pycnogonida taxa, and from Onychophora, *H. sapiens* and *C. elegans* as the outgroup species; after removal of low quality alignment regions, the supergene consists of 9,926 amino acid positions. In parallel, a set of 150 essential eukaryotic genes was extracted from 27 more distant species, including, mammals, amphibians, fish, plants, yeast and some arthropods. In this case, the resulting alignment consists of 23,685 amino acid positions. This approach had the aim of studying how the resulting topologies would be altered by adding the *C. noxius* coding sequences, and to analyze possible differential evolution rates within this species. The phylogenetic trees for both data sets were obtained with maximum likelihood, and they are shown in figure 2 (see Table S2 and Table S3 for full names of species considered in the concatenates).

Several conclusions can be addressed from the resulting trees. First of all, our topologies agree with the taxonomic classification of the organisms: in the arthropod specific context (Fig. 2B), *C. noxius* is grouped with *Hadrurus arizonensis* and *Heterometrus spinifer*, two scorpion species that belong to the Caraboctonidae and Scorpionidae families, respectively. Most of the tree bifurcations agree with the results described by Reiger and collaborators [20], however, our ML tree favours a more basal placement of Pycnogonida, instead of grouping Euchelicerata and sea spiders within the Chelicerata clade. Using 454 sequenced ESTs from the Emperor scorpion *Pandinus imperator*, Roeding and collaborators conducted a similar phylogenomic analysis in order to elucidate the evolutionary relationships between arthropods [32]. The tree topology derived in that report differs from the phylogeny shown in figure 2B in two aspects: (1) it suggests a closer relationship of Myriapoda and Chelicerata, whereas our study (as the one by [20]) strongly supports Mandibulata (Pancrustacea plus Myriapoda); (2) Pycnogonida species were included in the Euchelicerata clade. Some arthropod species showed long branches, but none of them corresponded to the included scorpion species, *P. imperator* and *M. gibbosus*, which had very poor sequence coverage (more than 70% missing positions in the alignment). This comparison reflects that the inferences that can be made from phylogenomic analyses are sensitive to the selection of data, although in general terms, congruent phylogenies can be recovered. In the eukaryotic data set (Fig. 2A), *C. noxius* is correctly grouped within the Arthropoda clade (that includes *D. melanogaster*, *A. aegipty* and *A. mellifera*), and the branch length comparison is in agreement with the divergence timing between different taxa: Chelicerata has a more basal placement over Hexapoda, mammals are closer in terms of evolutionary rates, and yeasts are placed at the bottom of the tree.

The second important consideration is that, in both cases, *C. noxius* displayed long-branch lengths that could reflect differential rates of evolution in some of the scorpion genes. In order to examine which where the coding sequences giving rise to this observation, the individual tree topologies of each gene were also analyzed. In most cases, the topologies supported with large bootstrap values the partitions between scorpions in the arthropod data set, and the arthropod partition in the eukaryotic dataset (Fig. S2). Other topologies however, grouped *C. noxius* with more distant organisms with low statistical support (Fig. S2). For both of these cases, the average branch length of the trees were calculated and compared to the branch length of *C. noxius* (Fig. S3 and Fig. S4). Even though important differences in branch lengths were expected to be associated to those topologies with taxonomic inconsistencies (which could have implied non-phylogenetic signal in the data), it was observed that *C. noxius* differed notably from the average tree length in only a few cases in both types of trees. Some examples of these divergent genes in the eukaryotic and arthropod specific data sets correspond to ribosomal proteins, aminoacyl-tRNA synthases, proteasome components and splicing factors (Fig. S3 and Fig. S4). It is also intriguing that most of these transcripts represent mitochondrial sequences. A previous report by Dávila and collaborators, in which the mitochondrial genome of *Centruroides limpidus* was sequenced and analyzed, revealed that this species has some important differences compared to *Mesobuthus gibbosus*, a buthid scorpion, and other spiders [33]. Particularly, the tRNAs showed structural characteristics (loops of variable sizes, poorly paired aminoacyl acceptor stems) that deviate from the classical cloverleaf structure. Together with these observations, it is possible that the variations in the branch lengths of the topologies we observed, might respond to different ribosomal features and tRNA positioning in the ribosomes that are particular to the genus *Centruroides*.

These gene-specific variations were buffered in the multigene analysis, showing that stochastic natural error naturally vanishes when more coding sequences are taken into account.

### Telson Specific Toxin Families

Several toxin families have been biochemically characterized in the venom of different Buthidae scorpions [1]. Among the most abundant and medically relevant toxins are the ionic-channel specific toxins, with different affinities for Na^+^, K^+^, Ca^2+^ and Cl^−^ channels. In the particular case of *C. noxius*, the sodium and potassium channel toxins have been described in our research group. They include some mammal-specific (Cn2–4 and Cn6–9) and insect-specific (Cn1, 5, 10–12) NaTxs [34], [35], the slotoxin, noxiustoxin, cobatoxin and Erg-toxins, these belonging to the KTx family [36]–[40].

The present study allowed us to identify other possible toxin types. Using blast results, signal peptide prediction and a phylogenetic analysis, which is a powerful strategy to address interesting questions not only related to the reconstruction of evolutionary relationships between species, but also, to predict the function of uncharacterized proteins and establish orthology and paralogy relationships between homologous genes, it was possible to obtain 72 different toxin-like isogroups. They represent only 0.4% of the total number of assembled transcripts and include ionic-channel specific toxins, antimicrobial peptides, enzymes, such as metalloproteases, hyaluronidase and phospholipases (Table 3), and other putative venom components whose function has not been described yet. Nevertheless, the presence of the corresponding peptides in the venom and their function will need to be addressed experimentally.

**Table 3:** Toxin families identified in *Centruroides noxius*.

Toxin Family			Isogroups	Reads	%identity	Known/New
**Ion channel specific toxins**	Sodium Channel*		27	7150	41–100%	12/22
	Potassium Channel*		15	9459	40–100%	10/8
	Calcium Channel		2	801	45–70%	0/2
	Others	LVP1	4	2845	31–45%	0/4
**Zinc Metalloproteases**	Antarease		7	5050	40–60%	0/7
	Astacin-like					
	VMP					
**Phospholipase**	PLA2		1	5	50%	0/1
**Protease inhibitors**	Kunitz-like		4	606	50–70%	0/4
**Serin-Proteases**	Salivary gland secreted Ser-Proteases		3	282	50%	0/3
**Lipase**	Colipase domain		1	230	60%	0/1
**Antimicrobial peptides**	Porine		1	6	60–78%	0/1
**Other venom components**	Venom insulin-like growth factor binding protein		2	2012	30–40%	0/2
	Hyaluronidase		1	15	70%	0/1
	Venom Allergen		1	26	66%	0/1
	Toxin-like peptide		1	1262	60%	0/1
	Neurotoxins		2	552	78%	0/2
**TOTAL**			72	30301		22/60

The isogroups are composed of telson specific reads.

* Toxin families previously characterized in the venom of this species. 22/27 NaTx and 8/15 KTx isogroups represent new putative toxin genes.

**Figure 3:**
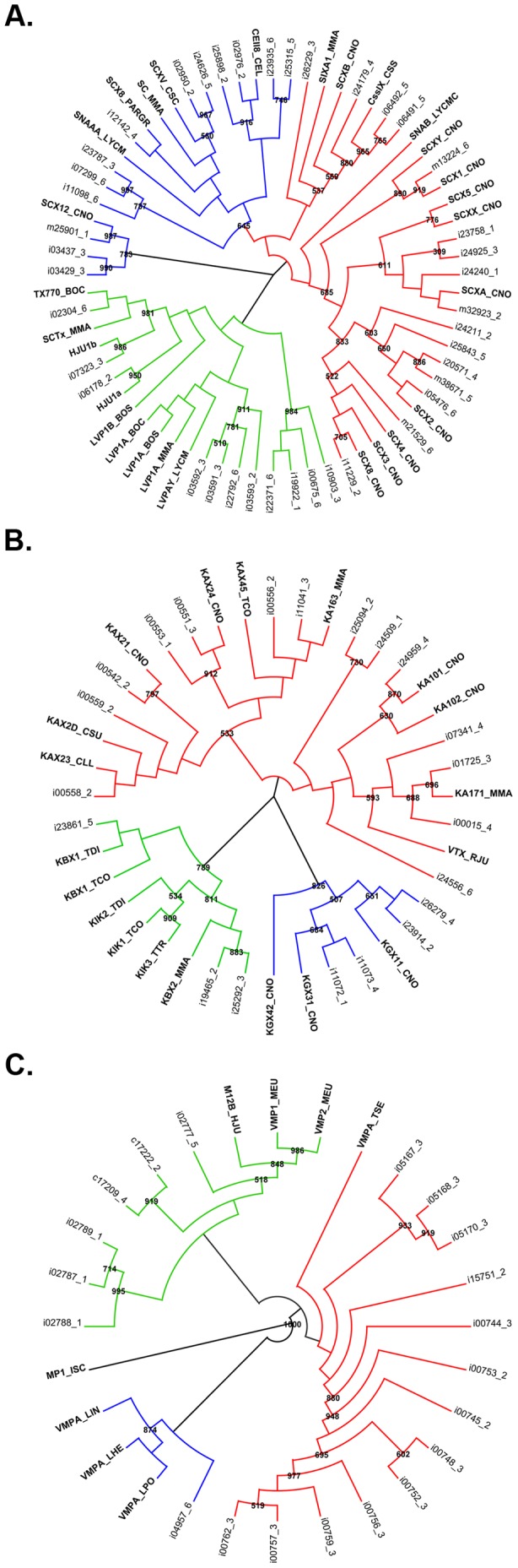
Maximum likelihood phylogenies of three different types of toxin-like transcripts from *C. noxius* and other scorpion/spider species taken from ToxProt (bolds). A. Sodium channel modifiers; beta type transcripts are highlighted in red, alpha type in blue and LVPs in green. B. Potassium channel blockers; alpha type transcripts are highlighted in red, beta type in green, gamma type in blue. C. Metalloproteases; venom metalloprotease-like isotigs are highlighted in green, astacin-like in blue and antarease-like in red. 1000 bootstrap pseudoreplicates were performed. Supported bifurcations (bootstrap values >500) are shown.

One of the most distinctive characteristics of Buthidae scorpions is the wide collection of NaTxs they express in their venom glands, which account for almost 10% of the protein content of some crude venoms (reviewed by [41]). This family can be divided into 2 different subfamilies according to the site of the channel they bind: alpha toxins (α-NaTx) bind voltage-independently at site-3 of sodium channels and inhibit the inactivation of the activated channels, thereby blocking neuronal transmission. On the other hand, beta toxins (β-NaTx) bind voltage-independently at site-4 of sodium channels and shift the voltage of activation toward more negative potentials thereby affecting sodium channel activation and promoting spontaneous and repetitive firing. In this study, 22 new putative NaTxs with variable identity values to other NaTxs of different species were obtained, and 5 of the already studied peptides were observed at the transcriptional level. From the phylogenetic analysis of these transcripts (Fig. 3A), several conclusions can be drawn. First, it is interesting to notice that even though the biochemical profile of *C. noxius* venom shows that only toxin Cn12 clearly belongs to the α subfamily [35], there are other assembled transcripts that are grouped in the α clade (blue branches) together with α-NaTxs from *C. sculpturatus, M. martensii, P. granulatus* and *L. mucronatus*. In the case of the β type, only the Cn1, 2, 4 and 10 toxins were obtained with identity values >90%, however, other branches in the topology showed assembled isotigs that are closely related to Cn5 and CngtII from *C. noxius*, CssIX from *C. suffusus suffusus*, CeII8 from *C. elegans* and Hj1a and Hj1b from *H. judaicus*. Another interesting clade of the topology is conformed by transcripts similar to lipolysis activating factors (LVPs), which share similarities with NaTx but display heterodimeric structures. These toxins were identified for the first time in the venom of *B. occitanus* [42], and were found to be abundant components of the venom of *L. mucronatus* [8]. Several isotigs were grouped in this clade (green branches of the topology), sharing 30–40% identity with LVPs form *B. occitanus, M. martensii* and *L. mucronatus*.

In contrast to the sodium channel specific toxins, those that block potassium channels showed less variability in their primary structure. Only the already known noxiustoxins and cobatoxins from the alpha subfamily and the Erg1 and 2 toxins from the gamma subfamily were successfully identified. However, from the phylogenetic analysis illustrated in figure 3B, it becomes clear that there are assembled transcripts similar to other α-KTx from *M. martensii, C. suffusus* and *T. costatus*, sharing from 36 to 92% identity. It was interesting as well to notice for the first time in a Centruroides species, the presence of beta-type KTx (scorpine-like and KIK toxins), which had only been described in the venoms of species from the families Scorpionidae, Caraboctonidae and several buthid genus, such as Tityus, Mesobuthus, Androctonus and Lychas [43]–[45]. This implies that β-KTxs are widely distributed in the Buthidae family.

In the above-mentioned cases (NaTxs and KTxs), it was not possible to detect the whole collection of toxins that are already known in this scorpion species but this is not a surprising observation. It has been reported that some major components of the crude venoms of scorpions such as *H. judaicus* [7] and *H. gerstchi* [46] cannot be detected at the transcriptional level, even after using specific primers for PCR amplification. In this regard, most of these peptides are stable due to the presence of disulfide bridges and other conformational characteristics, it is thus possible that once the genes are expressed and the toxins reach a certain concentration in the venom proteome, the genes are down-regulated and are no longer traceable with our experimental strategy. It is also possible that some of these transcripts undergo degradation directed by microRNAs, which is a common eukaryotic strategy to control RNA abundance and whose basic components were successfully identified in *C. noxius* as mention in previous sections. RNA extraction in shorter periods after scorpion milking might be useful to identify rapidly down-regulated genes that are not present at the time of collection of RNAs for cDNA libraries construction.

The phylogenetic analysis of metalloprotease-like transcripts (Fig. 3C) showed that there could be three different types of such enzymes in the venom of *C. noxius*. One of them, the antarease, has been recently described in another buthid scorpion, *Tityus serrulatus*, and its function seems to be related to the cleavage of v-SNARE proteins, affecting the correct vesicle trafficking in the cells [47]. In this study, five isogroups were found to share >50% identity with this enzyme; each isogroup was composed of several putative splicing variants that are highlighted in red in figure 3C. Whether these transcripts are different enzymes or just antarease isoforms will have to be validated. The second metalloprotease (green branch) was identified in the venom of *Mesobuthus eupeus* but has not been functionally characterized and, the last type is the astacin-like metalloproteases (blue branch). This has been identified in the venom of different *Loxosceles* spiders, and has also been implicated in the wrapping of silk fibbers in *Latrodectus* spiders [48]. The role during envenomations of this family of proteins has not been elucidated yet, however, it is possible that metalloproteases together with other enzymes such as hyaluronidases and protease inhibitors may work as diffusion factors for small neurotoxins; they might facilitate extra-oral digestion of preys or could also be used within the venom glands to process other venom components.

### Telson Differential Expression

The comparison of the transcriptional profile of the venom glands in resting state of a buthid scorpion, *H. judaicus*, and several other transcriptomic analyses performed collecting RNA from active venom glands (reviewed by [13]), revealed interesting features about toxin expression. Indeed, during resting states the transcript abundance of toxins appeared to be significantly reduced compared to other species from the same family in replenishing states; some low abundance decommissioned toxin-like transcripts (unlikely to be translated or secreted) were identified and intriguingly, the proliferation of protease transcripts was also observed. In this scenario, a huge amount of information regarding toxin diversity has been generated but has also restricted our knowledge on other cellular processes, metabolic pathways or regulatory networks that might have differential expression before and after venom extraction.

**Figure 4:**
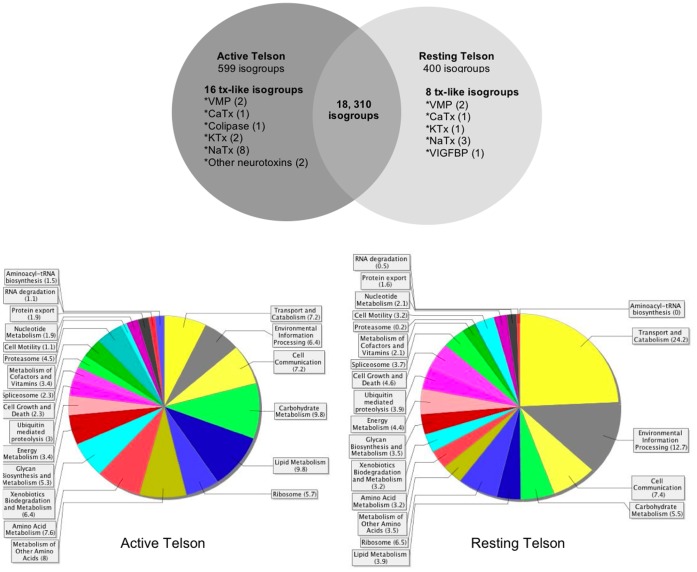
Differential transcriptional abundance observed in active and resting telson conditions. A. Number of isogroups showing differential expression validated with Fisher and Q tests (á<0,05). The intersection in the graph indicates those isogroups that are equally expressed in both conditions. B. Functional classification of the isogroups in A that have significant blast hits on NCBI-NR. The numbers in brackets represent the percentages over the total number of isogroups preferentially expressed in active or resting conditions.

The construction of cDNA libraries in two venom gland conditions of the same species, allowed us, for the first time, to directly compare the abundance of the whole transcriptional universe in the telson before and 5 days after venom extraction (Table S4). The statistical validation with Fisher and Q tests revealed that 3% and 2% of the isogroups were preferentially expressed during the active or resting states, respectively (Fig. 4A). Among these unique transcripts, 16 toxin-like isogroups were more abundant in the active state, whereas only 8 isogroups were more represented in the resting state. This result agrees with previous observations regarding the toxin profile in the venom glands, and indicates that the toxin genes expressed in resting states might contribute to the maintenance of basal concentrations of certain toxins in the venom. It was interesting to notice that none of the toxin-like isogroups with differential expression corresponded to the Cn2 toxin, the most toxic peptide of this species. The biological implications of these observations may be that, if Cn2 is such an important toxin for the scorpion survival then it needs to be rapidly restored in the venom and thus, five days after venom extraction the abundance of this transcript is sufficient to keep the required concentration of the protein. Another possibility is that given the fact that Cn2 has specificity for mammals and these are not common preys or predators of scorpions, the regeneration of this toxin is not a priority in the venom gland.

The rest of the isogroups with differential abundance that had similarity to other proteins in NR-NCBI, where mapped against the KEGG pathway database (Fig. 4B). In the active state, there is a high representation of carbohydrate, lipid and amino acid metabolism, a fact that corroborates the high metabolic cost of venom regeneration previously measured in *Parabuthus transvaalicus* [49]. A large proportion of proteasome components are also present in this condition, which might reflect that the venom gland needs a high quality control of the peptides it produces in order to attain its basal state. Considering venom extraction by electric stimulation as a source of environmental stress, it is not surprising to see the proteasome components highly expressed, since they are a good example of genes that are overexpressed under stress conditions, rather than repressed [50]. Additionally, it has to be mentioned that a growing number of recent studies have implicated some proteasome components in transcriptional regulation by establishing a specific protein interaction network of the transcription initiation factors at the upstream sequence of promoters that seems to be essential for the formation of the pre-initiation complex (both in proteolysis dependent and independent manners) [51], [52]. Genome-wide location analyses in yeast have revealed the proteasome’s preference for highly transcribed genes, including those involved in protein translation, glycolisis and lipid metabolism among others [51]. This regulatory capacity of the proteasome, together with the overexpression of its components and the KEGG categories preferentially expressed under telson activity, suggest that a similar mechanism could be taking place in the scorpion. Intriguingly, no transcription factors appear to be preferentially expressed under this telson condition however, the proteasome abundance could be overcoming this observation. Taking together the amino acid metabolism and the aminoacyl-tRNA biosynthesis categories, it is clear that there is an elevated translational activity, probably related to the regeneration of the peptide components of the venom. Under resting conditions (Fig. 4B), there is a higher representation of transcripts related to environmental information processing, which include membrane transport and signal transduction pathways. In contrast to the active state, under resting conditions there seems to be more endo/exocytosis events, peroxisomal and lysosomal activities, which are reflected in the high percentage of sequences under the transport and catabolism category.

Nevertheless, it is important to mention that 80% of the isogroups with differential transcriptional abundance had no significant identity to any other protein in NR-NCBI. This is an intriguing observation that indicates that important regulatory and functional differences might be given by genes of unknown function that should be carefully analyzed in the future.

### Conclusion

Even though the divergence timing of scorpions places them as interesting model organisms for evolutionary inferences, transcriptomic and proteomic studies performed over the years have mainly focused on the characterization of venom components. High-throughput sequencing platforms offer the possibility of generating thousands of sequences that have rapidly helped us to study the genomic organization of a wide range of species, and have substantially improved our understanding on phylogenetic and functional relationships between gene families. In this scenario, we took advantage of the 454 pyrosequencing system to analyze different aspects of the transcriptome of the Mexican scorpion *C. noxius*. Our sequencing and annotation strategies gave us a general view of the cellular and molecular processes that take place in these arthropods, revealing interesting features such as the presence of microRNAs and the small RNA processing machinery. It was possible to identify new toxin families specifically expressed in the telson of the scorpion that represent new pharmacological and biotechnological targets for future applications, and reflect the molecular complexity of the scorpion venoms. The phylogenomic analysis of concatenated coding genes uncovered important differences in evolution rates of specific sets of genes and, by means of a quantitative analysis of the transcriptional profiles of two different telson conditions (active and resting), several regulatory and metabolic responses were detected.

This massive effort to describe the molecular complexity of a scorpion species will need to be complemented with future studies at the genomic level, as well as functional validation of the gene families described along this report.

## Supporting Information

Figure S1 **Taxonomic profile of the transcriptome.** Arthropod specific sequences are highly represented by assembled isotigs (left), whereas some bacterial species are present among the singlets (right).
(TIFF)

Figure S2 **Bootstrap support of the scorpion (arthropod dataset) and arthropod (eukaryotic dataset) partitions in the individual tree topologies.** Positive percentages represent those topologies in which *C. noxius* was successfully grouped with other scorpions or arthropods; negative values imply that *C. noxius* was grouped with more distant organisms.
(TIFF)

Figure S3 **Branch length of the individual tree topologies from the arthropod dataset.** The average length and the branch length of *C. noxius* are indicated.
(TIFF)

Figure S4 **Branch length of the individual tree topologies from the eukaryotic dataset.** The average length and the branch length of *C. noxius* are indicated.
(TIFF)

Table S1 **Putative microRNAs.**
(DOC)

Table S2 **Eukaryotic species considered for the phylogenomic analysis shown in** **figure 2A** **.**
(DOC)

Table S3 **Arthropod species considered for the phylogenomic analysis shown in** **figure 2B** **.**
(DOC)

Table S4 **Read counts and expression specificity (ubiquitous, telson or body) for each isogroup.**
(XLS)
