# Supplementary figures and images for: Global Transcriptome Analysis of the Scorpion Centruroides noxius: New Toxin Families and Evolutionary Insights from an Ancestral Scorpion Species
Source: PLoS One. 2012 Aug 17;7(8):e43331. doi: 10.1371/journal.pone.0043331 (PMC3422302; doi:10.1371/journal.pone.0043331)

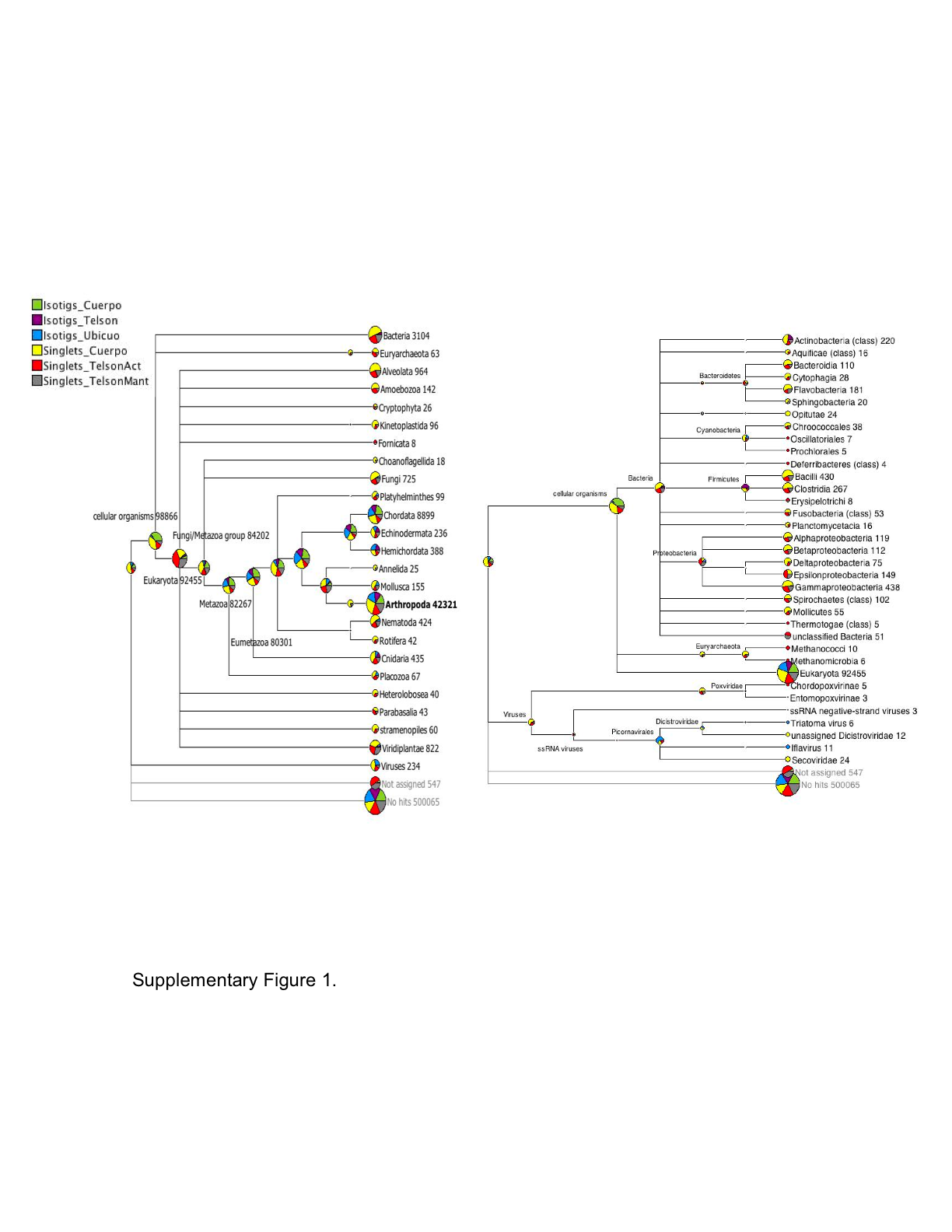

Supplement: Figure S1 — Taxonomic profile of the transcriptome. Arthropod specific sequences are highly represented by assembled isotigs (left), whereas some bacterial species are present among the singlets (right). (TIFF) [file pone.0043331.s001.tiff]

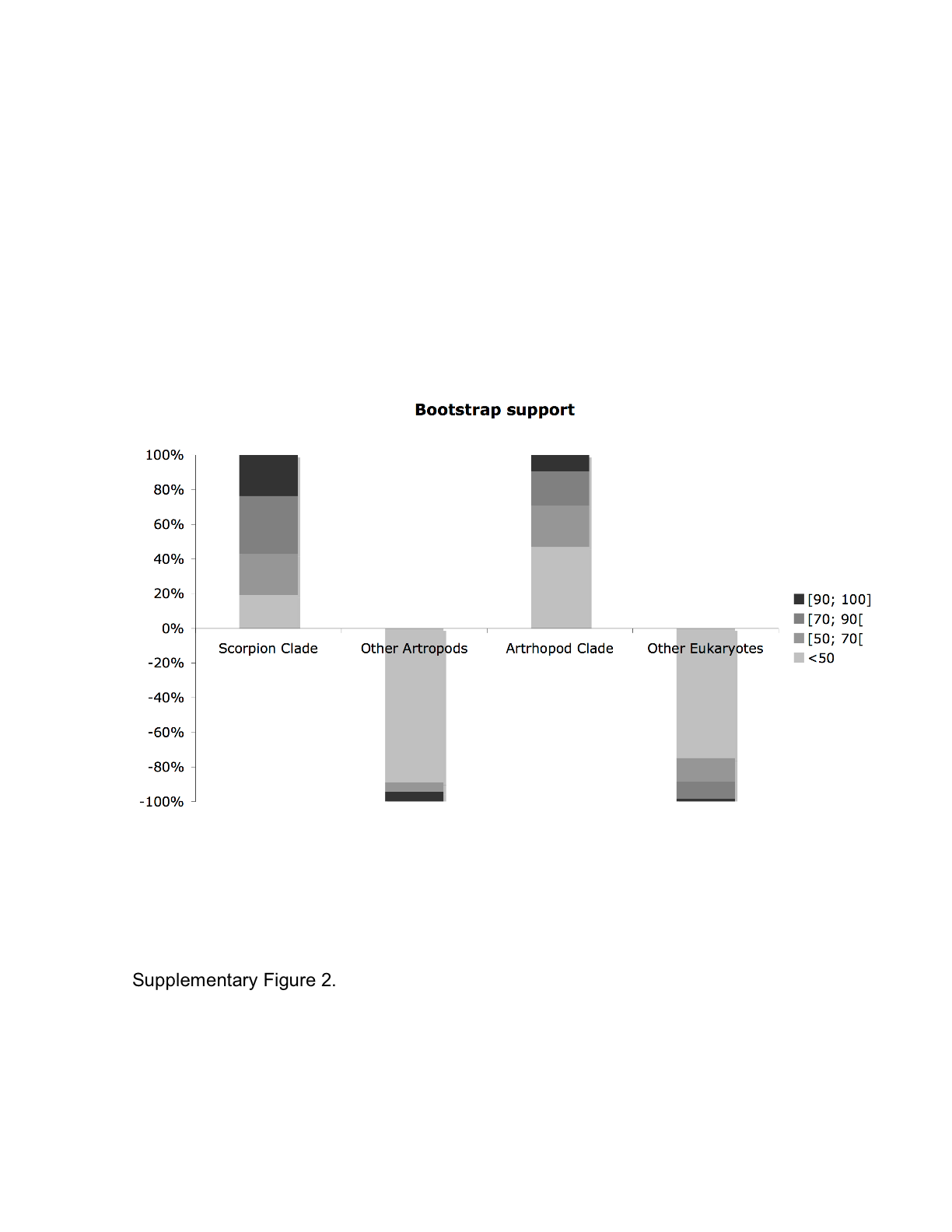

Supplement: Figure S2 — Bootstrap support of the scorpion (arthropod dataset) and arthropod (eukaryotic dataset) partitions in the individual tree topologies. Positive percentages represent those topologies in which C. noxius was successfully grouped with other scorpions or arthropods; negative values imply that C. noxius was grouped with more distant organisms. (TIFF) [file pone.0043331.s002.tiff]

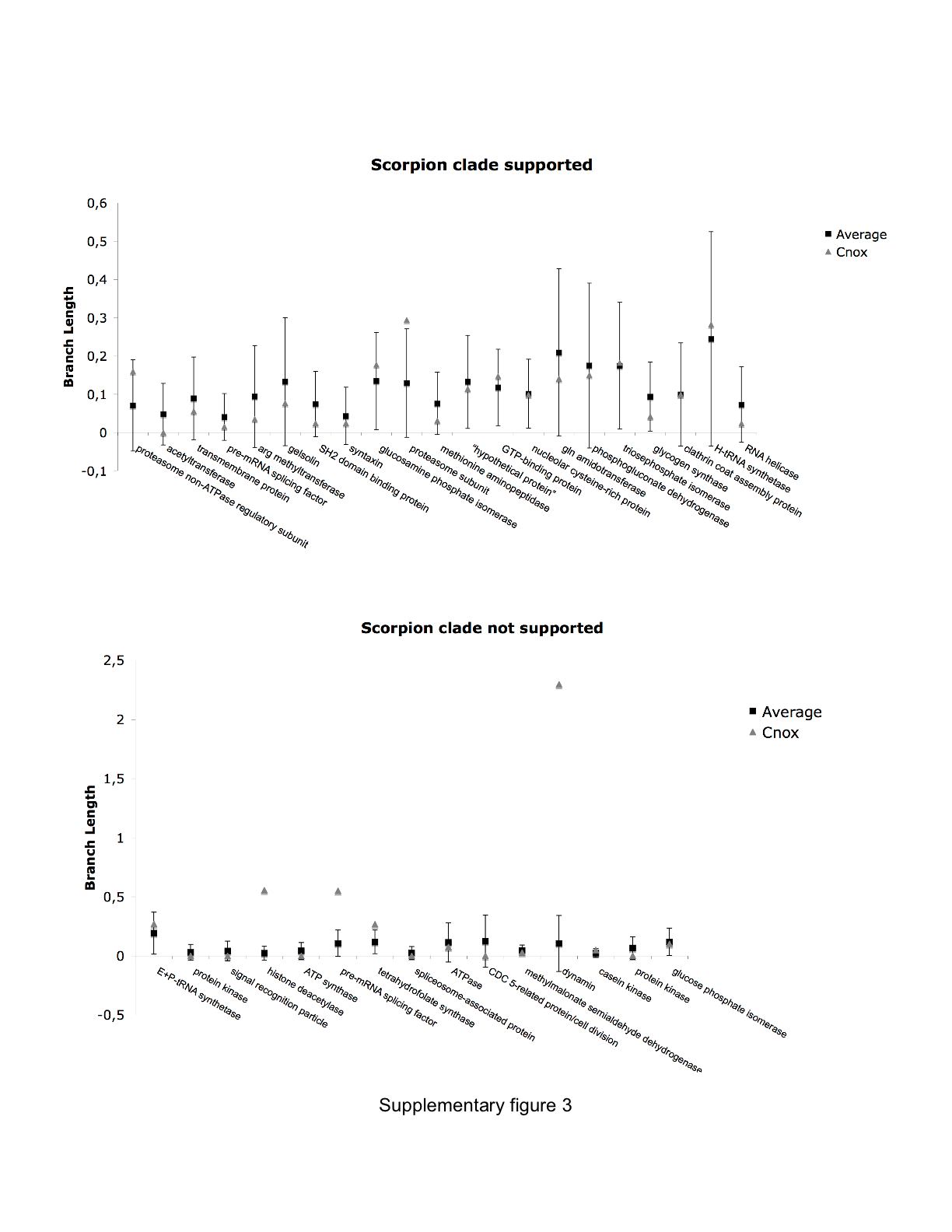

Supplement: Figure S3 — Branch length of the individual tree topologies from the arthropod dataset. The average length and the branch length of C. noxius are indicated. (TIFF) [file pone.0043331.s003.tiff]

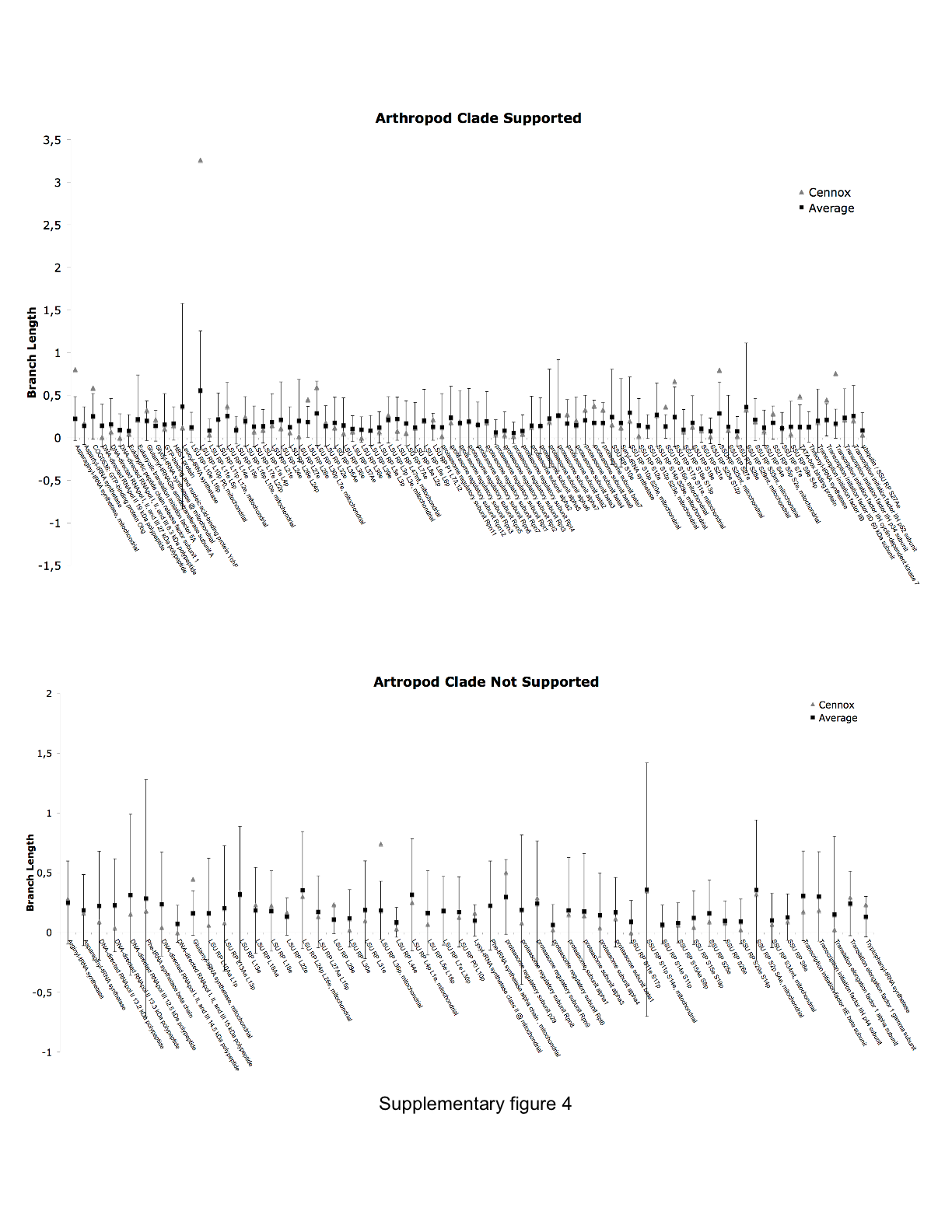

Supplement: Figure S4 — Branch length of the individual tree topologies from the eukaryotic dataset. The average length and the branch length of C. noxius are indicated. (TIFF) [file pone.0043331.s004.tif]
