# Supplementary material for: Global Transcriptome Analysis of the Scorpion Centruroides noxius: New Toxin Families and Evolutionary Insights from an Ancestral Scorpion Species
Source: PLoS One. 2012 Aug 17;7(8):e43331. doi: 10.1371/journal.pone.0043331 (PMC3422302; doi:10.1371/journal.pone.0043331)
Supplement: Table S1 — Putative microRNAs. (DOC) [file pone.0043331.s005.doc]

Supplementary table 1. Putative microRNAs.

| **Scorpion Sequence** | **microRNA-hairpin** | **%id** | **e-value** | **hairpin**  **coverage** | **mature microRNA** | **%id** | **Aln**  **bp** |
| --- | --- | --- | --- | --- | --- | --- | --- |
| GXU3QYM01A2OI8 | ppy-mir-566 | 83 | 5E-10 | 98,9 | hsa-miR-5689 | 100 | 12 |
| GXU3QYM01A2OI8 | ppy-mir-566 | 83 | 5E-10 | 98,9 | mml-miR-665 | 100 | 12 |
| GXU3QYM01A2OI8 | ppy-mir-566 | 83 | 5E-10 | 98,9 | ptr-miR-665 | 100 | 12 |
| GXU3QYM07H17KM | ppy-mir-566 | 87 | 4E-17 | 98,9 | hsa-miR-5585-3p | 100 | 17 |
| GXU3QYM07H17KM | ppy-mir-566 | 87 | 4E-17 | 98,9 | hsa-miR-5689 | 100 | 12 |
| GXU3QYM07H17KM | ppy-mir-566 | 87 | 4E-17 | 98,9 | bmo-miR-3296 | 100 | 12 |
| FXLSL4B01BPRPE | gga-mir-3533 | 86 | 2E-13 | 97,6 | rno-miR-196c | 100 | 14 |
| FXLSL4B01BPRPE | gga-mir-3533 | 86 | 2E-13 | 97,6 | ola-miR-196a | 100 | 11 |
| FXLSL4B01BPRPE | gga-mir-3533 | 86 | 2E-13 | 97,6 | aca-miR-196c | 100 | 11 |
| FXLSL4B01BPRPE | gga-mir-3533 | 86 | 2E-13 | 97,6 | aca-miR-196a | 100 | 11 |
| FXLSL4B01BPRPE | gga-mir-3533 | 86 | 2E-13 | 97,6 | pma-miR-196a | 100 | 11 |
| FXLSL4B01BPRPE | gga-mir-3533 | 86 | 2E-13 | 97,6 | ppy-miR-196b | 100 | 11 |
| isotig03213 | gga-mir-3533 | 86 | 1E-12 | 97,6 | rno-miR-196c | 100 | 14 |
| isotig03214 | gga-mir-3533 | 86 | 1E-12 | 97,6 | rno-miR-196c | 100 | 14 |
| isotig03215 | gga-mir-3533 | 86 | 9E-13 | 97,6 | rno-miR-196c | 100 | 14 |
| GXTJ2BI01A9Z1V | gga-mir-3533 | 85 | 5E-12 | 95,3 | rno-miR-196c | 100 | 14 |
| GXU3QYM03C4GL0 | ppy-mir-566 | 88 | 1E-19 | 94,5 | hsa-miR-1273f | 100 | 13 |
| GXU3QYM03C4GL0 | ppy-mir-566 | 88 | 1E-19 | 94,5 | bmo-miR-3296 | 100 | 12 |
| GXU3QYM03C4GL0 | ppy-mir-566 | 88 | 1E-19 | 94,5 | mml-miR-665 | 100 | 12 |
| GXU3QYM03C4GL0 | ppy-mir-566 | 88 | 1E-19 | 94,5 | ptr-miR-665 | 100 | 12 |
| GXU3QYM03C4GL0 | ppy-mir-566 | 88 | 1E-19 | 94,5 | hsa-miR-665 | 100 | 12 |
| FXCSSE001B2LKW | gga-mir-3533 | 89 | 2E-18 | 94,1 |  |  |  |
| GXTJ2BI07H1UZ8 | ppy-mir-1273 | 92 | 9E-30 | 91,3 | hsa-miR-1273g-3p | 100 | 16 |
| GXU3QYM07IB9UH | ppy-mir-1273 | 86 | 1E-17 | 91,3 | hsa-miR-1273f | 100 | 13 |
| GXU3QYM07IB9UH | ppy-mir-1273 | 86 | 1E-17 | 91,3 | hsa-miR-4459 | 100 | 13 |
| GXU3QYM07IB9UH | ppy-mir-1273 | 86 | 1E-17 | 91,3 | pma-miR-281a | 100 | 12 |
| GXTJ2BI04EIRF7 | hsa-mir-5096 | 91 | 4E-16 | 90,1 |  |  |  |
| GXU3QYM07H07VR | ppy-mir-1273 | 86 | 6E-16 | 88,5 | hsa-miR-1273g-3p | 94 | 18 |
| FXCSSE002HF4UG | hsa-mir-4426 | 87 | 9E-09 | 85,9 | gma-miR4374a | 100 | 11 |
| FXCSSE002HF4UG | hsa-mir-4426 | 87 | 9E-09 | 85,9 | ptr-miR-892b | 100 | 11 |
| GXU3QYM04EIZ03 | hsa-mir-4426 | 84 | 2E-04 | 85,9 |  |  |  |
| GXU3QYM08I2Z8D | ppy-mir-1273 | 89 | 5E-16 | 84,6 | hsa-miR-1273f | 95 | 19 |
| GXU3QYM08I2Z8D | ppy-mir-1273 | 89 | 5E-16 | 84,6 | hsa-miR-1273g-3p | 100 | 13 |
| GXU3QYM08I2Z8D | ppy-mir-1273 | 89 | 5E-16 | 84,6 | bfl-miR-4878 | 100 | 12 |
| FXLSL4B01EP7DB | hsa-mir-4426 | 83 | 2E-03 | 84,4 |  |  |  |
| EUC08LD02JBMAQ | hsa-mir-4426 | 87 | 1E-07 | 82,8 | gga-miR-1760 | 100 | 11 |
| FXCSSE002GKPFV | hsa-mir-4426 | 87 | 1E-07 | 82,8 | gga-miR-1760 | 100 | 11 |
| FXCSSE002IDD25 | hsa-mir-4426 | 83 | 4E-03 | 82,8 |  |  |  |
| FXLSL4B01C0CTZ | hsa-mir-4426 | 83 | 4E-03 | 82,8 |  |  |  |
| GXTJ2BI06GSQK5 | hsa-mir-4426 | 85 | 7E-05 | 82,8 |  |  |  |
| GXTJ2BI08JA1MY | hsa-mir-4426 | 85 | 7E-05 | 82,8 |  |  |  |
| GXU3QYM01BCG85 | hsa-mir-4426 | 87 | 2E-07 | 82,8 |  |  |  |
| GXU3QYM06GVLTI | hsa-mir-4426 | 83 | 4E-03 | 82,8 |  |  |  |
| GXU3QYM06G2YF3 | ppy-mir-1273 | 93 | 5E-26 | 81,7 | hsa-miR-1273f | 95 | 19 |
| GXU3QYM06G2YF3 | ppy-mir-1273 | 93 | 5E-26 | 81,7 | hsa-miR-1273g-3p | 94 | 17 |
| GXU3QYM06G2YF3 | ppy-mir-1273 | 93 | 5E-26 | 81,7 | mmu-miR-3070b-5p | 100 | 13 |
| GXU3QYM06G2YF3 | ppy-mir-1273 | 93 | 5E-26 | 81,7 | mmu-miR-3070a-5p | 100 | 13 |
| GXU3QYM07IMGYX | ppy-mir-1273 | 89 | 5E-16 | 80,8 | hsa-miR-1273f | 100 | 13 |
| GXU3QYM07IMGYX | ppy-mir-1273 | 89 | 5E-16 | 80,8 | mmu-miR-3070b-5p | 100 | 13 |
| GXU3QYM07IMGYX | ppy-mir-1273 | 89 | 5E-16 | 80,8 | mmu-miR-3070a-5p | 100 | 13 |
| GXU3QYM07IMGYX | ppy-mir-1273 | 89 | 5E-16 | 80,8 | gga-miR-1635 | 100 | 12 |
| GXU3QYM08JVFA2 | ppy-mir-1273 | 90 | 4E-23 | 80,8 | hsa-miR-1273e | 100 | 12 |
| EUC08LD01C1QZN | gga-mir-3533 | 85 | 2E-09 | 80 | hsa-miR-3171 | 100 | 11 |
| EUC08LD01C1QZN | gga-mir-3533 | 85 | 2E-09 | 80 | bta-miR-2346 | 100 | 11 |
| EUC08LD01C1QZN | gga-mir-3533 | 85 | 2E-09 | 80 | mmu-miR-1843-3p | 100 | 11 |
| EUC08LD01E36G2 | gga-mir-3533 | 85 | 2E-09 | 80 | hsa-miR-3171 | 100 | 11 |
| EUC08LD01E36G2 | gga-mir-3533 | 85 | 2E-09 | 80 | bta-miR-2346 | 100 | 11 |
| EUC08LD01E36G2 | gga-mir-3533 | 85 | 2E-09 | 80 | mmu-miR-1843-3p | 100 | 11 |
| GXTJ2BI03DI1NK | gga-mir-3533 | 87 | 2E-11 | 80 | rno-miR-196c | 100 | 14 |
| GXU3QYM02B2VL0 | gga-mir-3533 | 87 | 1E-11 | 80 | rno-miR-196c | 100 | 14 |
| GXU3QYM03C7TBM | gga-mir-3533 | 87 | 1E-11 | 80 | rno-miR-196c | 100 | 14 |
| GXTJ2BI01ATJ35 | hsa-mir-3927 | 98 | 3E-21 | 79,2 | hsa-miR-3927 | 100 | 22 |
| GXTJ2BI01ATJ35 | hsa-mir-3927 | 98 | 3E-21 | 79,2 | hsa-miR-4436a | 100 | 12 |
| GXTJ2BI01AUYP1 | gga-mir-3533 | 87 | 2E-11 | 78,8 | cin-miR-4082-3p | 100 | 11 |
| GXTJ2BI01AUYP1 | gga-mir-3533 | 87 | 2E-11 | 78,8 | cin-miR-4081-5p | 100 | 11 |
| GXTJ2BI01AUYP1 | gga-mir-3533 | 87 | 2E-11 | 78,8 | hsa-miR-3171 | 100 | 11 |
| GXTJ2BI01AUYP1 | gga-mir-3533 | 87 | 2E-11 | 78,8 | bmo-miR-2807b | 100 | 11 |
| GXU3QYM04EJ0ZG | gga-mir-3533 | 88 | 2E-13 | 78,8 |  |  |  |
| GXU3QYM07IEJPJ | ptr-mir-566 | 86 | 3E-12 | 78,7 | hsa-miR-1273f | 100 | 12 |
| ETFUWZZ01D92DM | hsa-mir-5096 | 85 | 7E-06 | 76,1 | tca-miR-3869-5p | 100 | 11 |
| ETFUWZZ01D92DM | hsa-mir-5096 | 85 | 7E-06 | 76,1 | mtr-miR169p | 100 | 11 |
| GXU3QYM02B5UX7 | hsa-mir-5096 | 83 | 3E-03 | 76,1 |  |  |  |
| ETFUWZZ02GED74 | ptr-mir-566 | 86 | 3E-11 | 75,5 | mml-miR-665 | 100 | 14 |
| ETFUWZZ02GED74 | ptr-mir-566 | 86 | 3E-11 | 75,5 | ptr-miR-665 | 100 | 14 |
| ETFUWZZ02GED74 | ptr-mir-566 | 86 | 3E-11 | 75,5 | hsa-miR-665 | 100 | 14 |
| ETFUWZZ02GED74 | ptr-mir-566 | 86 | 3E-11 | 75,5 | rno-miR-665 | 100 | 13 |
| ETFUWZZ02GED74 | ptr-mir-566 | 86 | 3E-11 | 75,5 | mmu-miR-665-3p | 100 | 13 |
| ETFUWZZ02GED74 | ptr-mir-566 | 86 | 3E-11 | 75,5 | gga-miR-1680 | 100 | 12 |
| ETFUWZZ02GED74 | ptr-mir-566 | 86 | 3E-11 | 75,5 | cin-miR-4207-3p | 100 | 11 |
| GXU3QYM06G5EV2 | ptr-mir-1273 | 87 | 2E-14 | 74,8 | hsa-miR-1273g-3p | 95 | 19 |
| GXU3QYM06G5EV2 | ptr-mir-1273 | 87 | 2E-14 | 74,8 | bfl-miR-2013 | 100 | 12 |
| GXU3QYM07IOBX1 | hsa-mir-5096 | 92 | 5E-12 | 74,6 |  |  |  |
| GXTJ2BI06GWIDK | ppy-mir-1273 | 90 | 3E-19 | 74,0 | hsa-miR-1273e | 100 | 12 |
| isotig21108 | nlo-mir-8 | 86 | 2E-07 | 73,6 |  |  |  |
| GXU3QYM03C5FWE | hsa-mir-5096 | 88 | 9E-09 | 73,2 |  |  |  |
| GXTJ2BI08JRSVZ | hsa-mir-1273a | 87 | 1E-10 | 72,1 | tca-miR-3828-5p | 100 | 12 |
| GXTJ2BI08JRSVZ | hsa-mir-1273a | 87 | 1E-10 | 72,1 | cel-miR-34-3p | 100 | 12 |
| GXTJ2BI01AVT46 | gga-mir-3533 | 90 | 2E-14 | 71,8 |  |  |  |
| EUKA23202H0XZD | isc-mir-96 | 89 | 9E-15 | 71,6 | isc-miR-96 | 95 | 22 |
| EUKA23202H0XZD | isc-mir-96 | 89 | 9E-15 | 71,6 | ppy-miR-96 | 95 | 21 |
| EUKA23202H0XZD | isc-mir-96 | 89 | 9E-15 | 71,6 | eca-miR-96 | 95 | 21 |
| EUKA23202H0XZD | isc-mir-96 | 89 | 9E-15 | 71,6 | dpu-miR-96 | 95 | 21 |
| EUKA23202H0XZD | isc-mir-96 | 89 | 9E-15 | 71,6 | cfa-miR-96 | 95 | 21 |
| EUKA23202H0XZD | isc-mir-96 | 89 | 9E-15 | 71,6 | bfl-miR-96 | 95 | 21 |
| EUKA23202H0XZD | isc-mir-96 | 89 | 9E-15 | 71,6 | bta-miR-96 | 95 | 21 |
| EUKA23202H0XZD | isc-mir-96 | 89 | 9E-15 | 71,6 | oan-miR-96 | 95 | 21 |
| EUKA23202H0XZD | isc-mir-96 | 89 | 9E-15 | 71,6 | mdo-miR-96 | 95 | 21 |
| EUKA23202H0XZD | isc-mir-96 | 89 | 9E-15 | 71,6 | xtr-miR-96 | 95 | 21 |
| GXTJ2BI08I53TP | gga-mir-3533 | 87 | 3E-07 | 70,6 | rno-miR-196c | 100 | 12 |
| GXTJ2BI08I53TP | gga-mir-3533 | 87 | 3E-07 | 70,6 | pvu-miR2119 | 100 | 11 |
| GXTJ2BI08I53TP | gga-mir-3533 | 87 | 3E-07 | 70,6 | gma-miR2119 | 100 | 11 |
| EUKA23201BPQOM | gga-mir-3533 | 92 | 5E-16 | 69,4 | cin-miR-4082-3p | 100 | 11 |
| EUKA23201BPQOM | gga-mir-3533 | 92 | 5E-16 | 69,4 | cin-miR-4081-5p | 100 | 11 |
| EUKA23201BPQOM | gga-mir-3533 | 92 | 5E-16 | 69,4 | bmo-miR-2807b | 100 | 11 |
| EUKA23202GP303 | gga-mir-3533 | 92 | 5E-16 | 69,4 | cin-miR-4082-3p | 100 | 11 |
| EUKA23202GP303 | gga-mir-3533 | 92 | 5E-16 | 69,4 | cin-miR-4081-5p | 100 | 11 |
| EUKA23202GP303 | gga-mir-3533 | 92 | 5E-16 | 69,4 | bmo-miR-2807b | 100 | 11 |
| GHXFOFM01A0XUM | gga-mir-3533 | 88 | 2E-11 | 69,4 | rno-miR-196c | 100 | 14 |
| GHXFOFM01A0XUM | gga-mir-3533 | 88 | 2E-11 | 69,4 | ola-miR-196a | 100 | 11 |
| GHXFOFM01A0XUM | gga-mir-3533 | 88 | 2E-11 | 69,4 | aca-miR-196c | 100 | 11 |
| GHXFOFM01A0XUM | gga-mir-3533 | 88 | 2E-11 | 69,4 | aca-miR-196a | 100 | 11 |
| GHXFOFM01A0XUM | gga-mir-3533 | 88 | 2E-11 | 69,4 | pma-miR-196a | 100 | 11 |
| GHXFOFM01A0XUM | gga-mir-3533 | 88 | 2E-11 | 69,4 | ppy-miR-196b | 100 | 11 |
| GHXFOFM01A0XUM | gga-mir-3533 | 88 | 2E-11 | 69,4 | ssc-miR-196b-5p | 100 | 11 |
| GHXFOFM01A0XUM | gga-mir-3533 | 88 | 2E-11 | 69,4 | eca-miR-196a | 100 | 11 |
| GHXFOFM01A0XUM | gga-mir-3533 | 88 | 2E-11 | 69,4 | eca-miR-196b | 100 | 11 |
| GHXFOFM01A0XUM | gga-mir-3533 | 88 | 2E-11 | 69,4 | bta-miR-196b | 100 | 11 |
| GXTJ2BI08JFN09 | gga-mir-3533 | 90 | 2E-11 | 69,4 | aca-miR-5408a* | 100 | 12 |
| GXU3QYM05FQ6UF | gga-mir-3533 | 88 | 2E-11 | 69,4 | rno-miR-196c | 100 | 14 |
| GXU3QYM05FQ6UF | gga-mir-3533 | 88 | 2E-11 | 69,4 | ola-miR-196a | 100 | 11 |
| GXU3QYM05FQ6UF | gga-mir-3533 | 88 | 2E-11 | 69,4 | aca-miR-196c | 100 | 11 |
| GXU3QYM05FQ6UF | gga-mir-3533 | 88 | 2E-11 | 69,4 | aca-miR-196a | 100 | 11 |
| GXU3QYM05FQ6UF | gga-mir-3533 | 88 | 2E-11 | 69,4 | pma-miR-196a | 100 | 11 |
| GXU3QYM05FQ6UF | gga-mir-3533 | 88 | 2E-11 | 69,4 | ppy-miR-196b | 100 | 11 |
| GXU3QYM05FQ6UF | gga-mir-3533 | 88 | 2E-11 | 69,4 | ssc-miR-196b-5p | 100 | 11 |
| GXU3QYM05FQ6UF | gga-mir-3533 | 88 | 2E-11 | 69,4 | eca-miR-196a | 100 | 11 |
| GXU3QYM05FQ6UF | gga-mir-3533 | 88 | 2E-11 | 69,4 | eca-miR-196b | 100 | 11 |
| GXU3QYM05FQ6UF | gga-mir-3533 | 88 | 2E-11 | 69,4 | bta-miR-196b | 100 | 11 |
| GXTJ2BI03DGWW8 | ptr-mir-1273 | 85 | 3E-08 | 68,9 | gga-miR-3532 | 100 | 12 |
| GXTJ2BI03DGWW8 | ptr-mir-1273 | 85 | 3E-08 | 68,9 | lgi-miR-1992 | 100 | 12 |
| GXTJ2BI03DGWW8 | ptr-mir-1273 | 85 | 3E-08 | 68,9 | cla-miR-1992 | 100 | 12 |
| GXTJ2BI03DGWW8 | ptr-mir-1273 | 85 | 3E-08 | 68,9 | cte-miR-1992 | 100 | 12 |
| EUKA23201B149O | isc-mir-96 | 87 | 1E-10 | 68,6 | isc-miR-96 | 95 | 22 |
| EUKA23201B149O | isc-mir-96 | 87 | 1E-10 | 68,6 | ppy-miR-96 | 95 | 21 |
| EUKA23201B149O | isc-mir-96 | 87 | 1E-10 | 68,6 | eca-miR-96 | 95 | 21 |
| EUKA23201B149O | isc-mir-96 | 87 | 1E-10 | 68,6 | dpu-miR-96 | 95 | 21 |
| EUKA23201B149O | isc-mir-96 | 87 | 1E-10 | 68,6 | cfa-miR-96 | 95 | 21 |
| EUKA23201B149O | isc-mir-96 | 87 | 1E-10 | 68,6 | bfl-miR-96 | 95 | 21 |
| EUKA23201B149O | isc-mir-96 | 87 | 1E-10 | 68,6 | bta-miR-96 | 95 | 21 |
| EUKA23201B149O | isc-mir-96 | 87 | 1E-10 | 68,6 | oan-miR-96 | 95 | 21 |
| EUKA23201B149O | isc-mir-96 | 87 | 1E-10 | 68,6 | mdo-miR-96 | 95 | 21 |
| EUKA23201B149O | isc-mir-96 | 87 | 1E-10 | 68,6 | xtr-miR-96 | 95 | 21 |
| GXTJ2BI03DMAEW | ppy-mir-1273 | 86 | 1E-10 | 68,3 | hsa-miR-1273e | 100 | 16 |
| GXTJ2BI03DMAEW | ppy-mir-1273 | 86 | 1E-10 | 68,3 | bfl-miR-2013 | 100 | 13 |
| EUKA23202JL9NO | ppy-mir-1273 | 93 | 1E-22 | 67,3 | hsa-miR-1273g-3p | 94 | 18 |
| EUKA23202JL9NO | ppy-mir-1273 | 93 | 1E-22 | 67,3 | hsv2-miR-H23 | 100 | 11 |
| GXU3QYM03C4HBG | gga-mir-3533 | 91 | 5E-15 | 67,1 | cin-miR-4082-3p | 100 | 11 |
| GXU3QYM03C4HBG | gga-mir-3533 | 91 | 5E-15 | 67,1 | cin-miR-4081-5p | 100 | 11 |
| GXU3QYM03C4HBG | gga-mir-3533 | 91 | 5E-15 | 67,1 | bmo-miR-2807b | 100 | 11 |
| EUC08LD01C4EUE | mmu-mir-1905 | 87 | 8E-09 | 66,3 | mcv-miR-M1-3p | 100 | 14 |
| EUC08LD01C4EUE | mmu-mir-1905 | 87 | 8E-09 | 66,3 | sme-miR-12* | 100 | 12 |
| GXTJ2BI08JJID5 | mmu-mir-1905 | 87 | 7E-09 | 66,3 | mcv-miR-M1-3p | 100 | 14 |
| GXTJ2BI08JJID5 | mmu-mir-1905 | 87 | 7E-09 | 66,3 | sme-miR-12* | 100 | 12 |
| EUC08LD02FM0S9 | gga-mir-3533 | 88 | 2E-09 | 65,9 |  |  |  |
| GXTJ2BI01BAHNE | hsa-mir-1273g | 83 | 3E-05 | 65,3 |  |  |  |
| GXU3QYM04EAST1 | ptr-mir-566 | 93 | 7E-19 | 64,9 | mml-miR-665 | 100 | 14 |
| GHXFOFM04EJ8NP | gga-mir-3533 | 89 | 4E-09 | 64,7 | hsa-miR-1273f | 100 | 11 |
| GXTJ2BI07IDIRG | pta-MIR1310 | 90 | 3E-11 | 64,2 |  |  |  |
| GXTJ2BI03C1AYM | gga-mir-3533 | 87 | 2E-08 | 63,5 | rno-miR-196c | 100 | 14 |
| GXTJ2BI03C1AYM | gga-mir-3533 | 87 | 2E-08 | 63,5 | ola-miR-196a | 100 | 11 |
| GXTJ2BI03C1AYM | gga-mir-3533 | 87 | 2E-08 | 63,5 | aca-miR-196c | 100 | 11 |
| GXTJ2BI03C1AYM | gga-mir-3533 | 87 | 2E-08 | 63,5 | aca-miR-196a | 100 | 11 |
| GXTJ2BI03C1AYM | gga-mir-3533 | 87 | 2E-08 | 63,5 | pma-miR-196a | 100 | 11 |
| GXTJ2BI03C1AYM | gga-mir-3533 | 87 | 2E-08 | 63,5 | ppy-miR-196b | 100 | 11 |
| GXTJ2BI03C1AYM | gga-mir-3533 | 87 | 2E-08 | 63,5 | ssc-miR-196b-5p | 100 | 11 |
| GXTJ2BI03C1AYM | gga-mir-3533 | 87 | 2E-08 | 63,5 | eca-miR-196a | 100 | 11 |
| GXTJ2BI03C1AYM | gga-mir-3533 | 87 | 2E-08 | 63,5 | eca-miR-196b | 100 | 11 |
| GXTJ2BI03C1AYM | gga-mir-3533 | 87 | 2E-08 | 63,5 | bta-miR-196b | 100 | 11 |
| isotig06511 | isc-mir-275 | 91 | 3E-13 | 62,7 | nlo-miR-275 | 96 | 23 |
| isotig06511 | isc-mir-275 | 91 | 3E-13 | 62,7 | ngi-miR-275 | 96 | 23 |
| isotig06511 | isc-mir-275 | 91 | 3E-13 | 62,7 | nvi-miR-275 | 96 | 23 |
| isotig06511 | isc-mir-275 | 91 | 3E-13 | 62,7 | isc-miR-275 | 96 | 23 |
| isotig06511 | isc-mir-275 | 91 | 3E-13 | 62,7 | dpu-miR-275 | 96 | 23 |
| isotig06511 | isc-mir-275 | 91 | 3E-13 | 62,7 | dya-miR-275 | 96 | 23 |
| isotig06511 | isc-mir-275 | 91 | 3E-13 | 62,7 | dwi-miR-275 | 96 | 23 |
| isotig06511 | isc-mir-275 | 91 | 3E-13 | 62,7 | dvi-miR-275 | 96 | 23 |
| isotig06511 | isc-mir-275 | 91 | 3E-13 | 62,7 | dsi-miR-275 | 96 | 23 |
| isotig06511 | isc-mir-275 | 91 | 3E-13 | 62,7 | dse-miR-275 | 96 | 23 |
| ETFUWZZ02I2K7K | gga-mir-3533 | 89 | 5E-10 | 62,4 | cin-miR-4082-3p | 100 | 11 |
| ETFUWZZ02I2K7K | gga-mir-3533 | 89 | 5E-10 | 62,4 | cin-miR-4081-5p | 100 | 11 |
| ETFUWZZ02I2K7K | gga-mir-3533 | 89 | 5E-10 | 62,4 | hsa-miR-3171 | 100 | 11 |
| ETFUWZZ02I2K7K | gga-mir-3533 | 89 | 5E-10 | 62,4 | bmo-miR-2807b | 100 | 11 |
| FXLSL4B01D1RBU | gga-mir-3533 | 89 | 3E-10 | 62,4 | cin-miR-4082-3p | 100 | 11 |
| FXLSL4B01D1RBU | gga-mir-3533 | 89 | 3E-10 | 62,4 | cin-miR-4081-5p | 100 | 11 |
| FXLSL4B01D1RBU | gga-mir-3533 | 89 | 3E-10 | 62,4 | hsa-miR-3171 | 100 | 11 |
| FXLSL4B01D1RBU | gga-mir-3533 | 89 | 3E-10 | 62,4 | bmo-miR-2807b | 100 | 11 |
| GXTJ2BI04EF5XJ | gga-mir-3533 | 87 | 4E-07 | 62,4 | rno-miR-196c | 100 | 14 |
| GXTJ2BI06HGKWJ | gga-mir-3533 | 87 | 7E-08 | 62,4 | rno-miR-196c | 100 | 14 |
| GXTJ2BI06HGKWJ | gga-mir-3533 | 87 | 7E-08 | 62,4 | ola-miR-196a | 100 | 11 |
| GXTJ2BI06HGKWJ | gga-mir-3533 | 87 | 7E-08 | 62,4 | aca-miR-196c | 100 | 11 |
| GXTJ2BI06HGKWJ | gga-mir-3533 | 87 | 7E-08 | 62,4 | aca-miR-196a | 100 | 11 |
| GXTJ2BI06HGKWJ | gga-mir-3533 | 87 | 7E-08 | 62,4 | pma-miR-196a | 100 | 11 |
| GXTJ2BI06HGKWJ | gga-mir-3533 | 87 | 7E-08 | 62,4 | ppy-miR-196b | 100 | 11 |
| GXTJ2BI06HGKWJ | gga-mir-3533 | 87 | 7E-08 | 62,4 | ssc-miR-196b-5p | 100 | 11 |
| GXTJ2BI06HGKWJ | gga-mir-3533 | 87 | 7E-08 | 62,4 | eca-miR-196a | 100 | 11 |
| GXTJ2BI06HGKWJ | gga-mir-3533 | 87 | 7E-08 | 62,4 | eca-miR-196b | 100 | 11 |
| GXTJ2BI06HGKWJ | gga-mir-3533 | 87 | 7E-08 | 62,4 | bta-miR-196b | 100 | 11 |
| GXTJ2BI07IAT4U | gga-mir-3533 | 87 | 7E-08 | 62,4 | rno-miR-196c | 100 | 14 |
| GXTJ2BI07IAT4U | gga-mir-3533 | 87 | 7E-08 | 62,4 | ola-miR-196a | 100 | 11 |
| GXTJ2BI07IAT4U | gga-mir-3533 | 87 | 7E-08 | 62,4 | aca-miR-196c | 100 | 11 |
| GXTJ2BI07IAT4U | gga-mir-3533 | 87 | 7E-08 | 62,4 | aca-miR-196a | 100 | 11 |
| GXTJ2BI07IAT4U | gga-mir-3533 | 87 | 7E-08 | 62,4 | pma-miR-196a | 100 | 11 |
| GXTJ2BI07IAT4U | gga-mir-3533 | 87 | 7E-08 | 62,4 | ppy-miR-196b | 100 | 11 |
| GXTJ2BI07IAT4U | gga-mir-3533 | 87 | 7E-08 | 62,4 | ssc-miR-196b-5p | 100 | 11 |
| GXTJ2BI07IAT4U | gga-mir-3533 | 87 | 7E-08 | 62,4 | eca-miR-196a | 100 | 11 |
| GXTJ2BI07IAT4U | gga-mir-3533 | 87 | 7E-08 | 62,4 | eca-miR-196b | 100 | 11 |
| GXTJ2BI07IAT4U | gga-mir-3533 | 87 | 7E-08 | 62,4 | bta-miR-196b | 100 | 11 |
| GXU3QYM03C9GQ3 | gga-mir-3533 | 89 | 3E-09 | 62,4 |  |  |  |
| GXU3QYM08JXE9H | gga-mir-3533 | 87 | 4E-07 | 62,4 | rno-miR-196c | 100 | 14 |
| GXU3QYM05GDROP | ptr-mir-566 | 88 | 7E-10 | 61,7 |  |  |  |
| EUKA23202HGC4Z | hsa-mir-1273g | 82 | 2E-03 | 61,4 | ath-miR5026 | 100 | 11 |
| EUKA23202HGC4Z | hsa-mir-1273g | 82 | 2E-03 | 61,4 | dme-miR-4985-5p | 100 | 11 |
| EUKA23202HGC4Z | hsa-mir-1273g | 82 | 2E-03 | 61,4 | ppy-miR-1285b | 100 | 11 |
| EUKA23202HGC4Z | hsa-mir-1273g | 82 | 2E-03 | 61,4 | bta-miR-2428 | 100 | 11 |
| EUKA23202HGC4Z | hsa-mir-1273g | 82 | 2E-03 | 61,4 | ptr-miR-1285 | 100 | 11 |
| EUKA23202HGC4Z | hsa-mir-1273g | 82 | 2E-03 | 61,4 | hsa-miR-1285-3p | 100 | 11 |
| FXLSL4B02JNAGD | gga-mir-3533 | 88 | 1E-09 | 61,2 | cin-miR-4082-3p | 100 | 11 |
| FXLSL4B02JNAGD | gga-mir-3533 | 88 | 1E-09 | 61,2 | cin-miR-4081-5p | 100 | 11 |
| FXLSL4B02JNAGD | gga-mir-3533 | 88 | 1E-09 | 61,2 | hsa-miR-3171 | 100 | 11 |
| FXLSL4B02JNAGD | gga-mir-3533 | 88 | 1E-09 | 61,2 | bmo-miR-2807b | 100 | 11 |
| GXU3QYM03CY9ZR | ptr-mir-1273 | 94 | 9E-20 | 60,2 | hsa-miR-1273g-3p | 100 | 18 |
| GXU3QYM04EHSQQ | hsa-mir-1273e | 92 | 3E-17 | 60,2 | hsa-miR-1273g-3p | 100 | 18 |
| GXU3QYM04EHSQQ | hsa-mir-1273e | 92 | 3E-17 | 60,2 | ppy-miR-1296 | 100 | 12 |
| EUC08LD02GII05 | gga-mir-3533 | 88 | 5E-09 | 60 | cin-miR-4082-3p | 100 | 11 |
| EUC08LD02GII05 | gga-mir-3533 | 88 | 5E-09 | 60 | cin-miR-4081-5p | 100 | 11 |
| EUC08LD02GII05 | gga-mir-3533 | 88 | 5E-09 | 60 | hsa-miR-3171 | 100 | 11 |
| EUC08LD02GII05 | gga-mir-3533 | 88 | 5E-09 | 60 | bmo-miR-2807b | 100 | 11 |
| GXTJ2BI08JH4DL | gga-mir-3533 | 86 | 9E-07 | 60 | rno-miR-196c | 100 | 14 |
| GXTJ2BI08JH4DL | gga-mir-3533 | 86 | 9E-07 | 60 | ola-miR-196a | 100 | 11 |
| GXTJ2BI08JH4DL | gga-mir-3533 | 86 | 9E-07 | 60 | aca-miR-196c | 100 | 11 |
| GXTJ2BI08JH4DL | gga-mir-3533 | 86 | 9E-07 | 60 | aca-miR-196a | 100 | 11 |
| GXTJ2BI08JH4DL | gga-mir-3533 | 86 | 9E-07 | 60 | pma-miR-196a | 100 | 11 |
| GXTJ2BI08JH4DL | gga-mir-3533 | 86 | 9E-07 | 60 | ppy-miR-196b | 100 | 11 |
| GXTJ2BI08JH4DL | gga-mir-3533 | 86 | 9E-07 | 60 | ssc-miR-196b-5p | 100 | 11 |
| GXTJ2BI08JH4DL | gga-mir-3533 | 86 | 9E-07 | 60 | eca-miR-196a | 100 | 11 |
| GXTJ2BI08JH4DL | gga-mir-3533 | 86 | 9E-07 | 60 | eca-miR-196b | 100 | 11 |
| GXTJ2BI08JH4DL | gga-mir-3533 | 86 | 9E-07 | 60 | bta-miR-196b | 100 | 11 |
| GXU3QYM03DFOW5 | hsa-mir-1273a | 89 | 6E-11 | 52,9 | hsa-miR-1273g-3p | 100 | 14 |
| GXU3QYM03DFOW5 | hsa-mir-1273a | 89 | 6E-11 | 52,9 | ssc-miR-30b-3p | 100 | 14 |
| GXU3QYM03DFOW5 | hsa-mir-1273a | 89 | 6E-11 | 52,9 | bta-miR-30b-3p | 100 | 14 |
| GXU3QYM03DFOW5 | hsa-mir-1273a | 89 | 6E-11 | 52,9 | hsa-miR-30b-3p | 100 | 14 |
| GXU3QYM03DFOW5 | hsa-mir-1273a | 89 | 6E-11 | 52,9 | hsa-miR-5197-5p | 94 | 16 |
| GXU3QYM03CY2DI | ppy-mir-1273 | 88 | 2E-06 | 49,0 | mghv-miR-M1-3* | 100 | 13 |
| GXU3QYM03CY2DI | ppy-mir-1273 | 88 | 2E-06 | 49,0 | aca-miR-30b | 100 | 11 |
| GXU3QYM03CY2DI | ppy-mir-1273 | 88 | 2E-06 | 49,0 | ppy-miR-30b | 100 | 11 |
| GXU3QYM03CY2DI | ppy-mir-1273 | 88 | 2E-06 | 49,0 | eca-miR-30b | 100 | 11 |
| GXU3QYM03CY2DI | ppy-mir-1273 | 88 | 2E-06 | 49,0 | ssc-miR-30b-5p | 100 | 11 |
| GXU3QYM03CY2DI | ppy-mir-1273 | 88 | 2E-06 | 49,0 | cfa-miR-30b | 100 | 11 |
| GXU3QYM03CY2DI | ppy-mir-1273 | 88 | 2E-06 | 49,0 | oan-miR-30b | 100 | 11 |
| GXU3QYM03CY2DI | ppy-mir-1273 | 88 | 2E-06 | 49,0 | xtr-miR-30b | 100 | 11 |
| GXU3QYM02CBZLK | ptr-mir-1273 | 92 | 2E-15 | 57,3 |  |  |  |
| GXU3QYM02BTMXN | hsa-mir-1273g | 83 | 2E-03 | 58,4 |  |  |  |
| GXTJ2BI08JPYN9 | ptr-mir-1273 | 88 | 8E-08 | 48,5 | hsa-miR-1273g-3p | 100 | 13 |
| GXTJ2BI08JH6MV | mmu-mir-1946a | 86 | 4E-03 | 37,0 |  |  |  |
| GXTJ2BI08I2G7U | sha-mir-716a | 89 | 2E-10 | 35,8 | rlcv-miR-rL1-24-5p | 100 | 12 |
| GXTJ2BI08I2G7U | sha-mir-716a | 89 | 2E-10 | 35,8 | aga-miR-87 | 100 | 12 |
| GXTJ2BI07HWTDJ | hsa-mir-1273g | 90 | 3E-10 | 49,5 | hsa-miR-1273g-3p | 94 | 18 |
| GXTJ2BI07HWTDJ | hsa-mir-1273g | 90 | 3E-10 | 49,5 | ppy-miR-1273 | 100 | 12 |
| GXTJ2BI06HK35T | ptr-mir-1273 | 86 | 4E-05 | 48,5 | hsa-miR-1273g-3p | 95 | 19 |
| GXTJ2BI06HK35T | ptr-mir-1273 | 86 | 4E-05 | 48,5 | gga-miR-1716 | 100 | 13 |
| GXTJ2BI04EO47M | ppy-mir-1273 | 85 | 2E-06 | 57,7 | bfl-miR-2013 | 100 | 12 |
| GXTJ2BI03C0BJT | peu-MIR2916 | 85 | 2E-04 | 55,9 |  |  |  |
| GXTJ2BI01A9FKZ | ptr-mir-1273 | 88 | 3E-08 | 49,5 | hsa-miR-1273g-3p | 100 | 14 |
| GXTJ2BI01A9FKZ | ptr-mir-1273 | 88 | 3E-08 | 49,5 | asu-miR-81b | 100 | 12 |
| EUKA23202HFB9G | ptr-mir-1273 | 88 | 2E-09 | 50,5 | ppy-miR-1296 | 100 | 12 |
| EUKA23202HFB9G | ptr-mir-1273 | 88 | 2E-09 | 50,5 | eca-miR-1296 | 100 | 12 |
| EUKA23202HFB9G | ptr-mir-1273 | 88 | 2E-09 | 50,5 | bta-miR-1296 | 100 | 12 |
| EUKA23202HFB9G | ptr-mir-1273 | 88 | 2E-09 | 50,5 | ptr-miR-1296 | 100 | 12 |
| EUKA23202HFB9G | ptr-mir-1273 | 88 | 2E-09 | 50,5 | hsa-miR-1296 | 100 | 12 |
| EUKA23202HFB9G | ptr-mir-1273 | 88 | 2E-09 | 50,5 | hsa-miR-4673 | 100 | 11 |
| EUKA23202HFB9G | ptr-mir-1273 | 88 | 2E-09 | 50,5 | pma-miR-4623 | 100 | 11 |
| EUKA23201ASUEV | mml-mir-619 | 90 | 1E-10 | 50 | rno-miR-665 | 100 | 12 |
| EUKA23201ASUEV | mml-mir-619 | 90 | 1E-10 | 50 | mmu-miR-665-3p | 100 | 12 |
| EUKA23201ASUEV | mml-mir-619 | 90 | 1E-10 | 50 | mml-miR-665 | 100 | 11 |
| EUKA23201ASUEV | mml-mir-619 | 90 | 1E-10 | 50 | ptr-miR-665 | 100 | 11 |
| EUKA23201ASUEV | mml-mir-619 | 90 | 1E-10 | 50 | hsa-miR-665 | 100 | 11 |
| EUKA23201ASUEV | mml-mir-619 | 90 | 1E-10 | 50 | cbr-miR-268 | 100 | 11 |
