# Supplementary material for: Global Transcriptome Analysis of the Scorpion Centruroides noxius: New Toxin Families and Evolutionary Insights from an Ancestral Scorpion Species
Source: PLoS One. 2012 Aug 17;7(8):e43331. doi: 10.1371/journal.pone.0043331 (PMC3422302; doi:10.1371/journal.pone.0043331)
Supplement: Table S2 — Eukaryotic species considered for the phylogenomic analysis shown in figure 2A . (DOC) [file pone.0043331.s006.doc]

Supplementary table 2. Eukaryote species considered for the phylogenomic analysis shown in figure 2A.

| **Eukaryote lineage** | **Species** |
| --- | --- |
| Amphibia | Xenopus tropicalis (Xen_tro) |
| Arthropoda | Anopheles gambiae (Ano_gam); Apis mellifera (Api_mel); Drosophila melanogaster (Dro_mel); Centruroides noxius (Cen_nox) |
| Aves | Gallus gallus (Gal_gal) |
| Fungi | Encephalitozoon cuniculi (Enc_cun); Eremothecium gossypii (Ere_gos); Gibberella zeae (Gib_zea); Magnaporthe grisea (Mag_gri); Neurospora crassa (Neu_cra); Saccharomyces cerevisiae (Sac_cer); Schizosaccharomyces pombe (Sch_pom) |
| Mammalia | Canis familiaris (Can_fam); Homo sapiens (Hom_sap); Mus musculus (Mus_mus); Pan troglodytes (Pan_tro); Rattus norvegicus (Rat_nor) |
| Nematoda | Caenorhabditis elegans (Cae_ele) |
| Teleostei | Danio rerio (Dan_rer); Fugu rubripes (Fug_rub); Tetraodon nigroviridis (Tet_nig) |
| Viridiplantae | Arabidopsis thaliana (Ara_tha); Oryza sativa (Ory_sat) |
| Others | Ciona intestinalis (Cio_int); Guillardia theta (Gui_the); Plasmodium falciparum (Pla_fal) |
