# Supplementary material for: Global Transcriptome Analysis of the Scorpion Centruroides noxius: New Toxin Families and Evolutionary Insights from an Ancestral Scorpion Species
Source: PLoS One. 2012 Aug 17;7(8):e43331. doi: 10.1371/journal.pone.0043331 (PMC3422302; doi:10.1371/journal.pone.0043331)
Supplement: Table S3 — Arthropod species considered for the phylogenomic analysis shown in figure 2B . (DOC) [file pone.0043331.s007.doc]

Supplementary table 3. Arthropod species considered for the phylogenomic analysis shown in figure 2B.

| **Lineage** | | | | **Species** |
| --- | --- | --- | --- | --- |
| **Arthropoda** | **Mandibulata** | **Pancrustacea** | **Hexapoda** | Podura aquatica (Paq); Orchesella imitari (Oim); Metajapyx subterraneus (Msu & Jap); Eumesocampa frigilis (Efr); Pedetontus saltator (Psa); Machiloides banksi (Mba); Ctenolepisma lineata (Cli); Nicoletia meinerti (Nme); Hexagenia limbata (May); Ephemerella inconstans (Ein); Ischnura verticalis (Ive); Libellula lydia (Lle); Periplaneta americana (Pam); Acheta domesticus (Ado) |
|  |  |  | **Crustacea** | Neogonodactylus oerstedii (Neo); Armadillidium vulgare (Avu); Libinia emarginata (Lem); Semibalanus balanoides (Bba); Chthamalus fragilis (Cfr); Lepas anserifera (Lean); Loxothylacus texanus (Lox); Acanthocyclops vernalis (A369); Eurytemora affinis (Eaf); Artemia salina (Asa3); Streptocephalus seali (ufs); Daphnia magna (Dma); Lynceus sp. (Lyn) |
|  |  |  | **Oligostraca** | Skogsbergia lerneri (Skle); Argulus sp. (Arg2); Armillifer armillatus (Aar) |
|  |  | **Myriapoda** | | Scutigera coleoptrata (Scol); Scolopendra polymorpha (Spo); Craterostigmus tasmanianus (Ctas); Abacion magnum (Ama); Polyzonium germanicum (Pge2); Polyxenus fasciculatus (Pol2); Hanseniella sp.(Han & Han2); Scutigerella sp.(Scu3); Eurypauropus spinosus (Eury) |
|  | **Chelicerata** | | **Arachnida & Merostomata** | Hadrurus arizonensis (Hari); Heterometrus spinifer (Hsp); Centruroides noxius (Cen_nox); Aphonopelma chalcodes (Ach); Parasitiformes: Amblyomma sp. (Amb2); Cryptocellus centralis (Crp); Dinothrombium pandorae (Din); Phrynus marginemaculatus (Pma); Stenochrus portoricensis (Stp); Eremocosta gigasella (Egig); Idiogaryops pumilis (Ipum); Prokoenenia wheeleri (Pwh); Carcinoscorpius rotundicauda (Cro) |
|  |  |  | **Pycnogonida** | Tanystylum orbiculare (Tor); Achelia echinata (Aeli); Endeis laevis (Ele); Ammothea hilgendorfi (Ahi) |
| **Onychophora** | | | | Euperipatoides rowelli (Erw & Ero); Peripatoides novaezealandiae (Pno2) |
| **Outgroups** | | | | Homo sapiens (Hsa); Caenorhabditis elegans (Cel) |
